# Supplementary material for: Planned, ongoing and completed tuberculosis treatment trials in Brazil, Russia, India, China and South Africa: a 2019 cross-sectional descriptive analysis
Source: BMJ Open. 2022 Jun 8;12(6):e057941. doi: 10.1136/bmjopen-2021-057941 (PMC9185397; doi:10.1136/bmjopen-2021-057941)
Supplement: Supplementary data [file bmjopen-2021-057941supp001.pdf]

Appendix 1

Search terms

"isoniazid", "rifampicin", "pyrazinamide", "ethambutol", "streptomycin", "rifabutin", "kanamycin", "amikacin", "capreomycin", "levofloxacin", "moxifloxacin", "ofloxacin", "para-aminosalicylic acid", "cycloserine", "terizidone", "ethionamide", "protionamide", "clofazimine", "linezolid", "amoxicillin/clavulanate", "thioacetazone", "imipenem/cilastatin", "high-dose isoniazid", "clarithromycin", "bedaquiline", "delamanid", "meropenem".

Coding of variables

**Type of intervention**
Drug therapy
Behavioral
Dots
Supplementation
Rehabilitation
Surgery

**Age of participants**	
Children	birth to 12 months
	1 to 5 years
	6 to 12 years
Adolescents only (<18)	13 to 17
Adults only	18 and over
Children and Adolescents	
Adolescents and adults	
All ages	
Not Reported	

**Ethics**
Local
International
Local and International
Not reported

**Funding sources**
Local Organisation
International organisation
Multiple funding
Industry
University
Hospital
Self funding
Unclear

**Co-morbidities**
HIV/AIDS
Diabetes
Rheumatoid disease
Multi-morbidity
Not applicable

**Type of drug treatment**
New drug
New combination
New treatment duration
New dosage
