# Supplementary material for: Planned, ongoing and completed tuberculosis treatment trials in Brazil, Russia, India, China and South Africa: a 2019 cross-sectional descriptive analysis
Source: BMJ Open. 2022 Jun 8;12(6):e057941. doi: 10.1136/bmjopen-2021-057941 (PMC9185397; doi:10.1136/bmjopen-2021-057941)
Supplement: Supplementary data [file bmjopen-2021-057941supp002.pdf]

Planned, ongoing and completed tuberculosis treatment trials in Brazil, Russia, India, China, and South Africa: A 2019 cross-sectional descriptive analysis

APPENDIX 2

SECTION A: Basic analysis

Table 1. Two-way table showing primary outcome reporting on trials completed 12 or more months by date versus registration status, recruiting country type, funding sources, and trial location.

	Primary outcome at completion	Primary outcome same for trial and published record	Primary outcome not the same for trial and published record	Total	P value
	**n(%)**	**n(%)**	**n(%)**	**No.**	
**Registration status**					
Not reported	7 (94)	0 (0)	1 (6)	8	0.001
Prospective	41 (38)	46 (43)	20 (19)	107	
Retrospective	7 (17)	24 (59)	10 (24)	41	
**Total**	**55 (35)**	**70 (45)**	**31 (20)**	**156**	
**Recruiting country type**					
Multi-national	42 (50)	35 (41)	8 (9)	85	0.0017
Single country	13 (18)	35 (49)	23 (32)	71	
**Total**	**55 (35)**	**70 (45)**	**31 (20)**	**156**	
**Funding source grouped**					
Local_Organisation	7 (17)	19 (48)	14 (35)	40	-
International_organisation	11 (28)	21 (52)	7 (18)	39	
Multifunding	3 (50)	3 (50)	0 (0)	6	
Industry	11 (39)	12 (43)	5 (18)	28	
University	3 (20)	7 (70)	1 (10)	10	
Hospital	6 (50)	3 (25)	3 (25)	12	
Self_funding	7 (70)	2 (20)	1 (10)	10	
Not_reported	7 (70)	3 (30)	0 (0)	10	
**Total**	**55 (35)**	**70 (45)**	**31 (20)**	**156**	
**Trial Location Country**					
Brazil	3 (60)	1 (20)	1 (20)	5	-
China	7 (64)	2 (13)	2 (13)	11	
India	2 (7)	17 (63)	8 (30)	27	
Multiple countries	22 (58)	10 (26)	6 (16)	38	
Russia	7 (27)	14 (54)	5 (19)	26	
South Africa	14 (29)	26 (53)	9 (18)	49	
**Total**	**55 (35)**	**70 (45)**	**31 (20)**	**156**	

Fisher exact test is used to calculate the p value. There is significant difference between registration status and primary outcome. There is also significance difference observed between recruitment

Planned, ongoing and completed tuberculosis treatment trials in Brazil, Russia, India, China, and South Africa: A 2019 cross-sectional descriptive analysis

country type and primary outcome. Funding source and trial location has small cell number, thus no p value provided.

Table 2. Two-way table comparing recruitment centres beween recruitment country types

Recruitment centres	Multiple countries		Single countries		Total		P value
	%	n	%	n	%	n	0.00
Multi-centre	56	77	44	59	100	136	
Single centre	0	0	98.3	117	100	119	
Total	30.4	77	69.6	176	100	253	

Fisher exact test is used to calculate the p value. There is significant difference when comparing recruitment centres between recruitment country types.

Table 3. Two-way table showing registration status versus trial location countries

Registration status	Brazil		China		India		Multiple countries		Russia		South Africa		Total	
	%	n	%	n	%	n	%	n	%	n	%	n	%	n
Not reported	0	0	0	0	0	0	0	0	91.3	21	0	0	8.3	21
Prospective	36.4	4	69.1	38	50.9	27	85.7	66	4.3	1	79.4	27	64.4	163
Retrospective	63.6	7	30.9	17	49.1	26	14.3	11	4.3	1	20.6	7	27.3	69
Total	100	11	100	55	100	53	100	77	100	23	100	34	100	253

Table 3: Two-way table comparing registration status between recruiting country type

Registration status	Multiple countries		Single countries		Total		P value
	%	n	%	n	%	n	0.00
Not reported	0	0	11.9	21	8.3	21	
Prospective	85.7	66	55.1	97	64.4	163	
Retrospective	14.3	11	33	58	27.3	69	
Total	100	77	100	176	100	253	

Fisher exact test is used to calculate the p value. There is significant difference when comparing registration status between recruitment country type.

Table 4: Two-way table comparing recruitment status between recruitment country types

Recruitment status	Multiple countries		Single countries		Total		P value
	%	No.	%	No.	%	No.	0.039
Complete	1.3	1	0.6	1	0.8	2	
Not recruiting	70.1	54	64.2	113	66	167	
Not reported	2.6	2	11.9	21	9.1	23	
Recruiting	24.7	19	23.3	41	23.7	60	
Terminated	1.3	1	0	0	0.4	1	
Total	100	77	100	176	100	253	

Planned, ongoing and completed tuberculosis treatment trials in Brazil, Russia, India, China, and South Africa: A 2019 cross-sectional descriptive analysis

Fisher exact test is used to calculate the p value. There is significant difference when comparing recruitment status between recruitment country type.

Table 6: Two-way table comparing funding sources between recruitment country types

Funding source grouped	Multiple countries		Single countries		Total		P value*
	%	No.	%	No.	%	No.	0.00
**Local_Organisation**	11.7	9	31.3	55	25.3	64	
**International_organisation**	46.8	36	12.5	22	22.9	58	
**multifunding**	9.1	7	6.8	12	7.5	19	
**Industry**	20.8	16	15.9	28	17.4	44	
**University**	5.2	4	10.2	18	8.7	22	
**Hospital**	1.3	1	7.4	13	5.5	14	
**Self_funding**	1.3	1	8	14	5.9	15	
**Other**	3.9	3	8	14	6.7	17	
**Total**	100	77	100	176	100	253	

*chi 2 used to calculate the p value. There are significant differences when comparing funding sources between recruitment country types.

Table 7: Two-way table comparing primary outcomes of published trials between recruitment country types.

Primary Outcome	Multiple countries		Single countries		Total		P value
	%	No.	%	No.	%	No.	0.029
**Yes**	81.4	35	60.3	35	69.3	70	
**No**	18.6	8	39.7	23	30.7	31	
**Total**	100	43	100	58	100	101	

Fisher exact test is used to calculate the p value. There is significant difference when comparing primary outcome of published studies only between recruitment country type.

Planned, ongoing and completed tuberculosis treatment trials in Brazil, Russia, India, China, and South Africa: A 2019 cross-sectional descriptive analysis

SECTION B: Survival analysis.

This graph shows the overall Kaplan-Meier survival estimate, with a median publication time of 25 months.

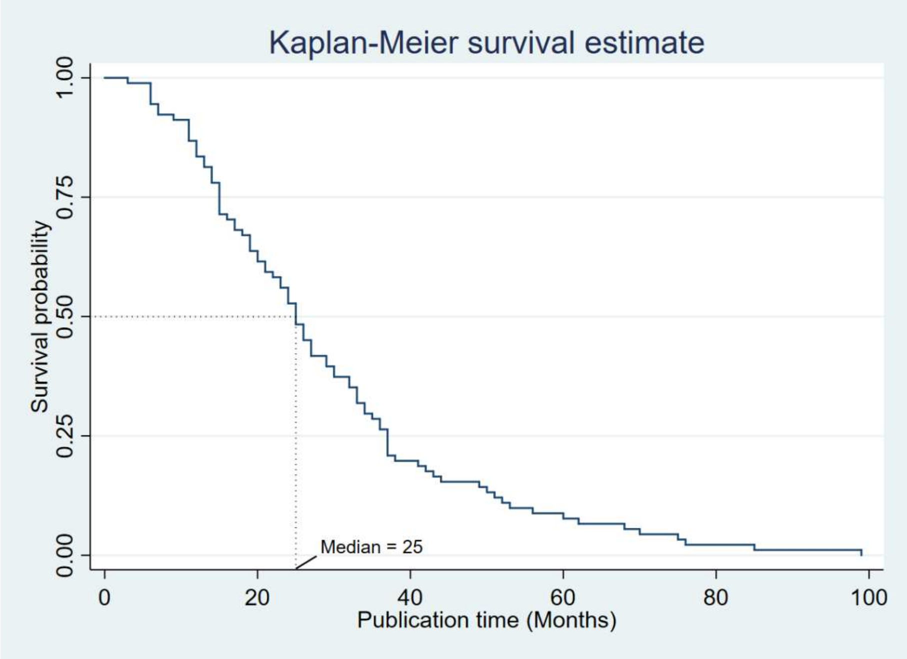

Figure 1: This graph shows the overall Kaplan-Meier survival estimate for single country with a median publication time of 26 months.

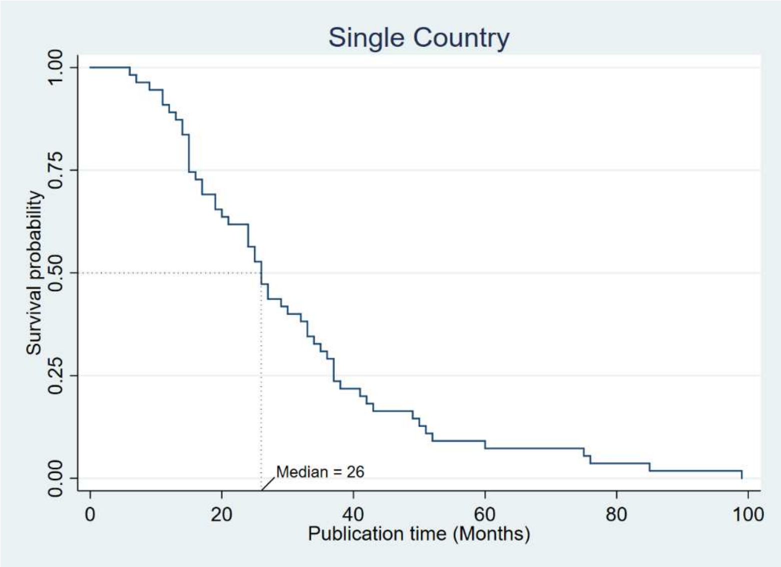

Planned, ongoing and completed tuberculosis treatment trials in Brazil, Russia, India, China, and South Africa: A 2019 cross-sectional descriptive analysis

Figure 2: This graph shows the overall Kaplan-Meier survival estimate for multinational studies with a median publication time of 23 months. This shows that multinational studies takes shorter time to publish as compared with single country study.

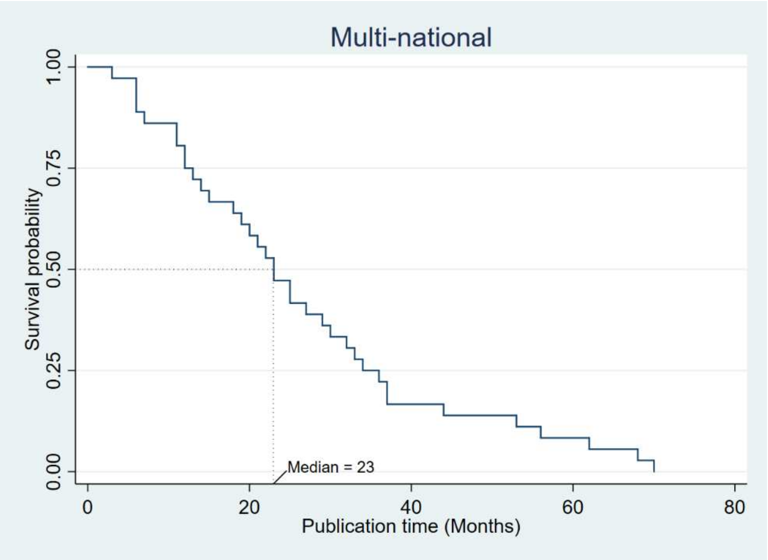

Figure 3: This graph shows the overall Kaplan-Meier survival estimate for India trial location with a median publication time of 29 months.

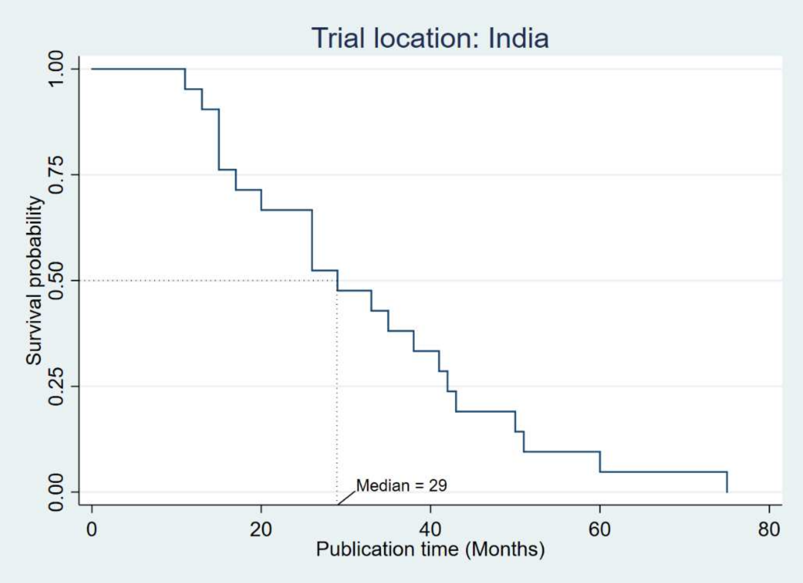

Planned, ongoing and completed tuberculosis treatment trials in Brazil, Russia, India, China, and South Africa: A 2019 cross-sectional descriptive analysis

Figure 4: This graph shows the overall Kaplan-Meier survival estimate for China trial location with a median publication time of 25 months.

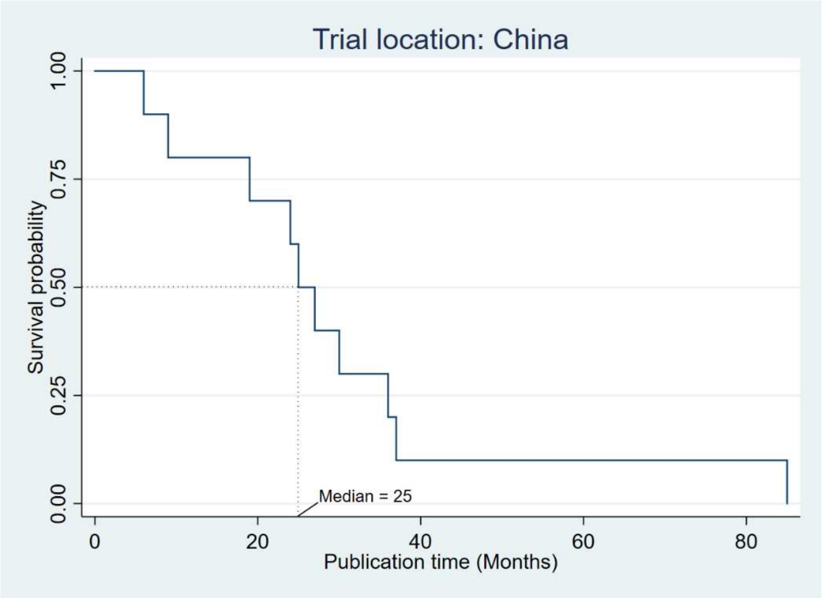

Figure 5: This graph shows the overall Kaplan-Meier survival estimate for Multi-countries trial location with a median publication time of 23 months.

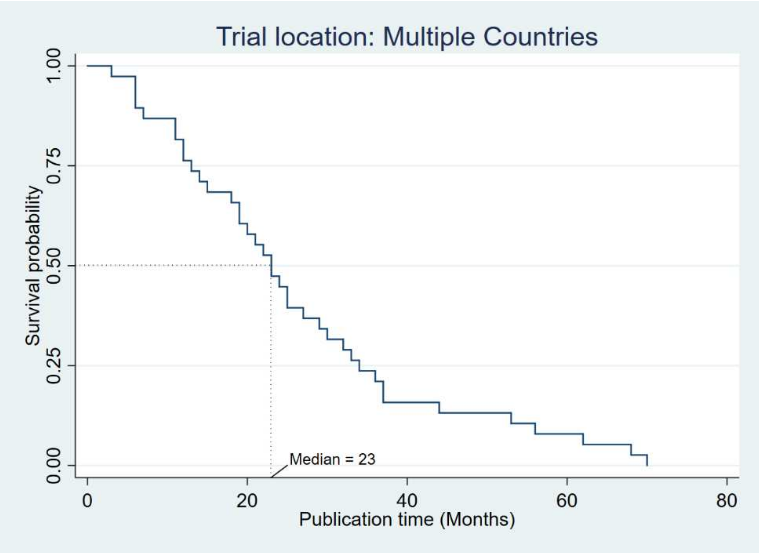

Figure 6: This graph shows the overall Kaplan-Meier survival estimate for combine Brazil, Russia and South Africa trial location with a median publication time of 24 months.

Planned, ongoing and completed tuberculosis treatment trials in Brazil, Russia, India, China, and South Africa: A 2019 cross-sectional descriptive analysis

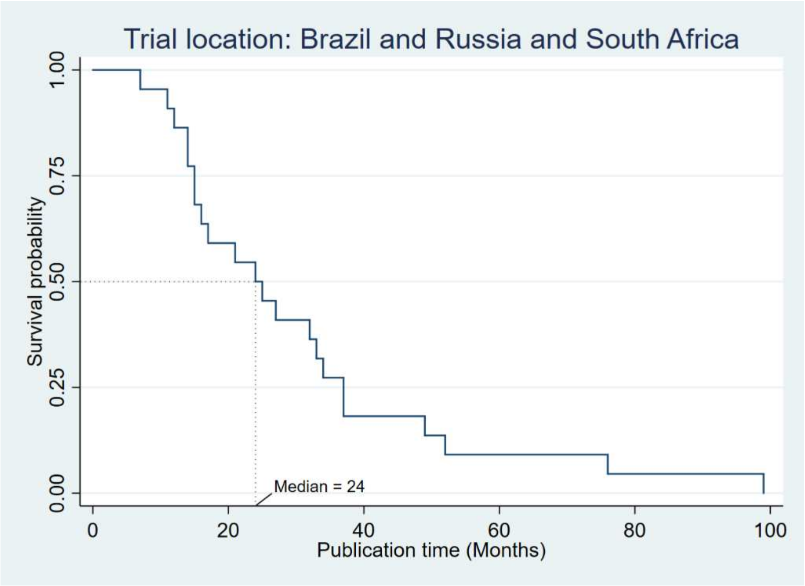

Figure 7: Multi-countries trial location studies takes shorter time to publish as compared to the other trial locations.

Planned, ongoing and completed tuberculosis treatment trials in Brazil, Russia, India, China, and South Africa: A 2019 cross-sectional descriptive analysis

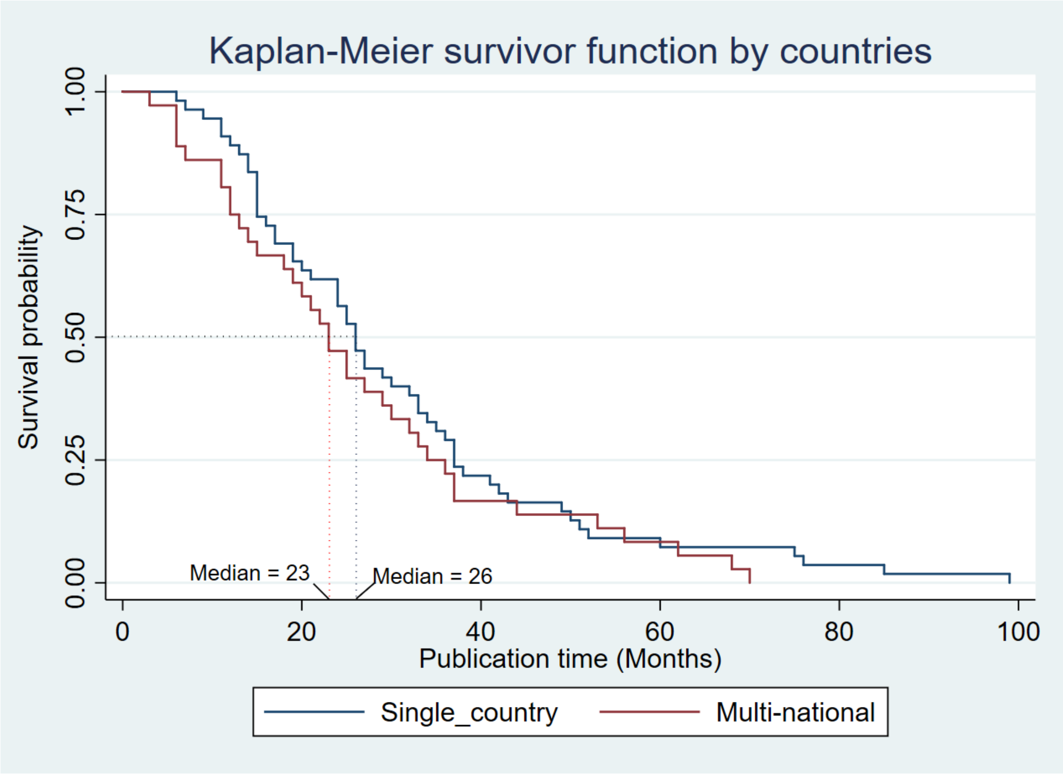

Figure 8: graph showing publication time for single country verse multi-national country.

Table 8: Test For Equality Between Groups

	n (%)	Logrank test	Wilcoxon test
Countries			
Single	171 (70)	0.3117	0.2617
Multi-national	73 (30)		
Trial country			
China	52 (21)	0.6789	0.5012
India	51 (21)		
Multiple Countries	73 (30)		
Brazil, Russia & SA	68 (28)		
Registrationstat			
Not reported	9 (21)	0.413	0.3558
Prospective	64 (156)		
Retrospective	27 (67)		

BMJ Publishing Group Limited (BMJ) disclaims all liability and responsibility arising from any reliance placed on this supplemental material which has been supplied by the author(s)

Planned, ongoing and completed tuberculosis treatment trials in Brazil, Russia, India, China, and South Africa: A 2019 cross-sectional descriptive analysis

The table 1, compares the curves between the group for each variable used for survival analysis. We used Log-rank test and Wilcoxon test to compares whether the publication time between the groups is different in average. We see that there is no significance difference between the groups. For example, there is no significance difference between publication time for single country verse multi-national country.
