# Supplementary material for: Planned, ongoing and completed tuberculosis treatment trials in Brazil, Russia, India, China and South Africa: a 2019 cross-sectional descriptive analysis
Source: BMJ Open. 2022 Jun 8;12(6):e057941. doi: 10.1136/bmjopen-2021-057941 (PMC9185397; doi:10.1136/bmjopen-2021-057941)
Supplement: Author's manuscript [file bmjopen-2021-057941.draft_revisions.pdf]

BMJ Open

BMJ Open is committed to open peer review. As part of this commitment we make the peer review history of every article we publish publicly available.

When an article is published we post the peer reviewers' comments and the authors' responses online. We also post the versions of the paper that were used during peer review. These are the versions that the peer review comments apply to.

The versions of the paper that follow are the versions that were submitted during the peer review process. They are not the versions of record or the final published versions. They should not be cited or distributed as the published version of this manuscript.

BMJ Open is an open access journal and the full, final, typeset and author-corrected version of record of the manuscript is available on our site with no access controls, subscription charges or pay-per-view fees (http://bmjopen.bmj.com).

If you have any questions on BMJ Open's open peer review process please email info.bmjopen@bmj.com

Planned, ongoing and completed tuberculosis treatment trials in Brazil, Russia, India, China, and South Africa: A 2019 cross-sectional descriptive analysis

Journal:	*BMJ Open*
Manuscript ID	bmjopen-2021-057941
Article Type:	Original research
Date Submitted by the Author:	04-Oct-2021
Complete List of Authors:	Mathebula, Lindi; South African Medical Research Council; Stellenbosch University, Department of Global Health<br>Mapahla, Lovemore; Stellenbosch University, Department of Global Health<br>Nurkhametova, Dilyara; Russian Medical Academy for Continuing Professional Education of the Ministry of Health; 4. A.I. Virtanen Institute, University of Eastern Finland, Kuopio, Finland<br>Ziganshina, Liliya Eugenevna; Russian Medical Academy for Continuing Professional Education of the Ministry of Health<br>Mazinu, Mikateko; Medical Research Council of South Africa<br>Jordan, Esme; South African Medical Research Council; University of the Western Cape, Statistics and Population Studies Department<br>Ndwandwe, Duduzile; South African Medical Research Council, Cochrane South Africa<br>Kredo, Tamara; South African Medical Research Council, South African Cochrane Centre
Keywords:	Tuberculosis < INFECTIOUS DISEASES, Clinical trials < THERAPEUTICS, Respiratory infections < THORACIC MEDICINE

SCHOLARONE™ Manuscripts

I, the Submitting Author has the right to grant and does grant on behalf of all authors of the Work (as defined in the below author licence), an exclusive licence and/or a non-exclusive licence for contributions from authors who are: i) UK Crown employees; ii) where BMJ has agreed a CC-BY licence shall apply, and/or iii) in accordance with the terms applicable for US Federal Government officers or employees acting as part of their official duties; on a worldwide, perpetual, irrevocable, royalty-free basis to BMJ Publishing Group Ltd ("BMJ") its licensees and where the relevant Journal is co-owned by BMJ to the co-owners of the Journal, to publish the Work in this journal and any other BMJ products and to exploit all rights, as set out in our licence.

The Submitting Author accepts and understands that any supply made under these terms is made by BMJ to the Submitting Author unless you are acting as an employee on behalf of your employer or a postgraduate student of an affiliated institution which is paying any applicable article publishing charge ("APC") for Open Access articles. Where the Submitting Author wishes to make the Work available on an Open Access basis (and intends to pay the relevant APC), the terms of reuse of such Open Access shall be governed by a Creative Commons licence – details of these licences and which Creative Commons licence will apply to this Work are set out in our licence referred to above.

Other than as permitted in any relevant BMJ Author's Self Archiving Policies, I confirm this Work has not been accepted for publication elsewhere, is not being considered for publication elsewhere and does not duplicate material already published. I confirm all authors consent to publication of this Work and authorise the granting of this licence.

For peer review only

Submission for BMJ Open

Planned, ongoing and completed tuberculosis treatment trials in Brazil, Russia, India, China, and South Africa: A 2019 cross-sectional descriptive analysis

Lindi Mathebula[1,2*], Lovemore Mapahla[2], Dilyara Nurkhametova[3,4], Liliya Eugenevna Ziganshina[3], Mikateko Mazinu[5], Esme Jordan[5,6], Duduzile ndwandwe[1], Tamara Kredo[1]

1. Cochrane South Africa, South African Medical Research Council, Tygerberg, Cape Town, South Africa
2. Division of Epidemiology and Biostatistics, Department of Global Health, Stellenbosch University, Cape Town, South Africa
3. Cochrane Russia, Russian Medical Academy for Continuing Professional Education of the Ministry of Health, Moscow, Russian Federation and St Albans House, 57-59 Haymarket, London, United Kingdom
4. A.I. Virtanen Institute, University of Eastern Finland, Kuopio, Finland
5. Biostatistics unit, South African Medical Research Council, Tygerberg, Cape Town, South Africa
6. Statistics and Population Studies Department, University of the Western Cape

* Corresponding author

E-mail: lindi.mathebula@mrc.ac.za

Keywords: *Tuberculosis, Treatment, Trials, BRICS, Primary registries*

For peer review only

Submission for BMJ Open

ABSTRACT

Tuberculosis (TB) remains a deadly challenge globally and Brazil, Russia, India, China, and South Africa (BRICS) are amongst the countries with the highest TB burden. The objective of this study is to identify and describe ongoing, planned, and completed TB trials conducted in the BRICS countries registered in World Health Organisation International Clinical Trial Registry (WHO-ICTRP); to report selective outcome reporting by comparing primary outcomes in published trials with their pre-specified outcomes in registry records and to evaluate the time to publication.

Methods and analysis: We searched the WHO-ICTRP portal (20 January 2019) and the Russian Federation Registry (30 March 2019) to identify TB trials conducted in BRICS countries. We included only registered clinical trials conducted wholly in BRICS countries or with at least one recruitment centre in one of the BRICS countries that were investigating TB treatment.

Results: The search of the WHO-ICTRP yielded 408 trials and additional 32 trials were identified from the Russian registry. Of those, 253 were included in the analysis. We found that 77 trials were multi-country trials, followed by trials in China (55), India (53), South Africa (34), Russia (23), and Brazil (11). One hundred and sixty-three (163) trials were registered prospectively, 69 retrospectively, and 21 trials had no registration status. Most trials (207) evaluated TB treatment, followed by 29 behaviour change interventions, 13 nutritional supplementation, four surgical treatment and two assessing rehabilitation. Thirty-one of the 101 trials with publication had evidence of selective outcome reporting. The median time to publication was 25 months (IQR 15-37) from the time of anticipated end date stated in the registry.

Conclusion: TB trials conducted in BRICS countries are collaborative, mostly drug treatment oriented, potentially affecting policies. Selective outcome reporting remains a problem both for prospectively and retrospectively registered trials, only small fraction of which gets to publication.

For peer review only - http://bmjopen.bmj.com/site/about/guidelines.xhtml

STRENGTHS AND LIMITATIONS OF THIS STUDY

- This study addresses a research gap by describing and analysing planned, ongoing and completed TB treatment trials specific to middle-income countries with high TB burden, Brazil, Russia, India, China and South Africa.
- We conducted an extensive search of relevant clinical trials registries including the WHO-ICTRP and the Russian Trials Registry to ensure that no publicly available trial is missed.
- The selection of trials and data extraction were conducted in duplicate to minimise bias and ensure rigor.
- Since initial search on the 20th of January 2021, there have been 20 more publication from included clinical trials. Studies published after our analysis are unlikely to change the description analysis we have performed.
- As not all trials are registered or submitted to the WHO-ICTRP, some trials may have been missed.

For peer review only

INTRODUCTION

Tuberculosis (TB) remains one of the most challenging and deadliest infectious diseases in the world ranking as the ninth cause of death worldwide [1-2]. Although TB mortality and incidence have been reported to be decreasing by 3% and 2% respectively, most countries have been lagging behind in meeting the United Nations 2030 Sustainable Development Goal of ending the TB epidemic [2]. Brazil, Russia, India, China, and South Africa, (BRICS) countries, have been reported to have a very high burden of TB, hosting 46% of all global TB cases and 40% of TB related deaths [3]. Furthermore, antimicrobial resistance has led to increasing cases of Multi-Drug Resistant TB (MDR-TB) of which China, India, and Russia account for more than half (56%) of the global burden [3]. In addition to the Sustainable Development Goals launched in 2015 aiming to end TB by 2030; the World Health Organization (WHO) launched a strategy in 2014 called the WHO's End TB strategy, which is endorsed by the World Health Assembly, to eradicate the global TB epidemic by 2035 [3-4].

The WHO's End TB 2035 strategy aims to decrease TB related deaths by at least 95% and an overall 90% reduction in TB incidence to 10 cases/100 000 or fewer with TB-affected households experiencing no costs of TB related care [5]. One of the three pillars of the WHO End TB strategy is research and innovation. While research studies regularly estimate incidence, mortality rates and TB management approaches, new innovations of treatments are often not easily tracked [2]. To track TB research efforts, Lienhardt et al suggested a strategic plan that included mapping research funding involvement to determine where the research funding gaps are [6]. Another component of this plan is a Global TB Research Roadmap, which identified research gaps that should be closed in order to stop TB [6]. A study by Bai et al (2018) aimed to conduct a bibliometric analysis of research and development (R&D) using publications to find out what contributions the BRICS countries make in the area for neglected tropical diseases including TB [7]. Another approach to identify research activity in different areas suggested by Viergever et al is to use data from registered trials in primary clinical trials registries [8].

Mapping clinical trial activity through clinical trial registries provides an insight into where most research has been conducted and what type of research is being done or is planned and ongoing [8-10]. Clinical trials are primary research methods that are undertaken to study effects of new interventions such as treatment [11]. Randomised controlled trials (RCTs) have

For peer review only - http://bmjopen.bmj.com/site/about/guidelines.xhtml

been described as the gold standard study design to inform policy and practice decisions regarding what interventions work better for improving healthcare [12]. However, studies showed that 37% of clinical trials presented in abstract and registered in trial registries never reach publication [12-13]. Furthermore, those that reach publication often omit negative results and deviate from pre-specified outcomes leading to publication bias [12-14]. A systematic review looking at quality of outcome reporting in phase 2 studies of TB treatment showed evidence that these studies often had variations between reported outcomes and trial characteristics [15]. Research has suggested that for trials to be transparent, trialists should register their trials prospectively in a public platform indicating the aim of the study and the outcomes to be measured before the trial begins, to minimise selective outcome reporting [16].

Primary registries are online platforms that register and make public planned clinical trials and the data from different primary registries is aggregated and also made publicly available in the WHO International Clinical Trial Registry Platform (WHO-ICTRP) [17]. This then creates the necessary public accountability mechanism to ensure that planned trials and their results cannot be hidden regardless of their outcomes, which reduces the temptation to not publish results that are negative [18]. Prospective registration of trial in a primary registry means registration of a trial before it starts and is now widely recognized as a key strategy to increase research transparency. Prospective registration is supported by the Consolidated Standards of Reporting Trials (CONSORT) Statement and The International Committee of Medical Journals Editors (ICMJE), which includes many of the world's leading journals [18-20]. Retrospective registration of a trial means a trial that is registered after the first participant has been enrolled into the trial and is not a recommended practice as it diminishes research integrity according to the ICJME and CONSORT statement [18-20].

BRICS countries have become major contributors to clinical research worldwide. Funding for research in the TB field and the number of publications has been seen to increase on a yearly basis having almost doubled in the past decade [21]. Research shows there is a likelihood of omitting or deviating from outcomes or measurement of outcomes when results are not favorable in trials including TB trials [14-15]. This may lead to overestimation of the efficacy of interventions and lead to adverse outcomes when implemented into policy. Proehl et al (2018) has indicated the need for transparency in clinical trials from trial registration to

For peer review only - http://bmjopen.bmj.com/site/about/guidelines.xhtml

publication in order to effect a positive change in policy [22]. In light of this, the objective of this study is to identify and describe ongoing, planned, and completed TB trials conducted in the BRICS countries registered in WHO-ICTRP and report any selective outcome reporting by identifying registered trials that have been published to compare reported outcomes with those pre-specified in the trial registration. Mapping TB treatment trials will assist researchers to identify potential research gaps, sponsors and funders to identify funding gaps, patients to identify available treatments, policy makers to identify evidence-based interventions to end TB epidemic.

OBJECTIVES

The main objective of the study is to identify and describe ongoing, planned, and completed TB trials conducted in the BRICS countries registered in WHO-ICTRP and report any selective outcome reporting by identifying registered trials that have been published to compare reported outcomes with those pre-specified in the trial registration.

Hypothesis

The hypothesis of this study is that there is a difference between outcomes specified trial publications and those pre-specified in the trial registration.

MATERIALS AND METHODS

Study Sample

We aimed to include all registered trials planned for conduct in BRICS countries from inception of the WHO-ICTRP data repository in 2005 to the date of search. We searched the WHO-ICTRP portal on 20 January 2019 to identify TB trials conducted in BRICS countries using the following search terms: “tuberculosis” AND “Treatment” OR “Therapy” OR “Management” AND “Country-name” i.e each of the BRICS countries. Two researchers Lindi Mathebula (LIM) and Lovemore Maphahla (LOM) independently screened all records of the search and selected trials to be included. The Russian Federation does not have a primary registry that submits trials data to the WHO-ICTRP platform. Therefore, two researchers Dilyara Nurkhametova (DN) and Liliya Eugenevna Ziganshina (LEZ) from Cochrane Russia (based at Russian Medical Academy for Continuing Professional Education, the Russian Federation), searched the Russian trial registry, named the State Registry of Medicines (GRLS,

For peer review only - http://bmjopen.bmj.com/site/about/guidelines.xhtml

For peer review only

grls.rosminzdrav.ru) (30 March 2019). This registry contains trial information on all clinical trials officially approved by the Federal Ministry of health. Since the Russian registry does not allow for an advanced search with Boolean operators, we searched for trials using the term "tuberculosis" followed by a series of separate searches for each of the following antituberculosis medicines (search terms available in appendix 1).

We included only registered clinical trials regardless of status of the trial (i.e. ongoing, planned, or completed) conducted wholly in BRICS countries or with at least one recruitment centre in one of the BRICS countries that were investigating interventions for the treatment of TB. Trials were either randomised controlled trials, controlled clinical trials including cluster trials, or multiple-arm trials. Upon retrieval of the search output, we removed duplicates from the sample and excluded trials that were not eligible. WHO-ICTRP considers the first trial record registered to take precedence for trials registered in more than one primary register, however we considered the trial record with the latest date and excluded the other records to ensure that we have a full record of the changes that were made from earlier trial registration.

After selection of the trials into the study, two researchers (LIM, LOM) independently used trial identification numbers to search for publications in PubMed and Cochrane Library. DN and LEZ searched the Russian electronic database of research publications eLIBRARY.RU using trial identification numbers and antituberculous medicines names. Publications were further identified in the trial record under the field "URL to publication" which is one of the 24 data items that is collected by WHO-ICTRP primary registries.

Data extraction

Trial records

We downloaded data from the WHO-ICTRP and imported into Microsoft Excel™ with the following data items: registry name, trial identification, initial application date, start date of trial, end date of trial, date of registration, registration status (retrospective/prospective), principal investigator, source of funding, type of treatment, primary and secondary outcomes, inclusion/exclusion criteria, age of participants, and sample size amongst other baseline characteristics. Data from the Russian trial registry was manually entered in the excel format under the same data items. Two researchers (LIM, LOM) independently reviewed data from

For peer review only - http://bmjopen.bmj.com/site/about/guidelines.xhtml

the WHO-ICTRP manually and extracted further data points from individual records of included trials, similarly two researchers (LEZ, DN) from Russia independently and manually reviewed data from the Russian trial registry and extracted further data points from individual records. The researchers translated records that were in Russian language to English before sending data in Excel format.

Publication extraction

Two researchers (LIM, LOM) independently extracted data from all published studies of the included trials in Excel for comparison. The following data items were extracted from the publication: title of the study, publication date, trial identification listed (yes/no), study design, age of participants, sample size, primary outcomes. We combined the data extracted by the two researchers from South Africa and two researchers from the Russian Federation for cleaning and coding. For selective outcome reporting we compared what was reported in the trial record as a primary outcome including the type of outcome and time points for the measurement of the outcome, to what was reported in the publication as a primary outcome. We used "yes" if the authors reported the same outcome and "no" if they reported a different primary outcome in the publication. We coded the data using variables in categories that unified the data (Appendix 1).

Data Analysis

Once entered data was reviewed and cleaned to ensure that no data was missing, the completed and cleaned data set was imported into the StataCorp. 2015 for statistical analysis. All data was stored on the South African Medical Research Council network and backed up on a daily basis to ensure no loss of original data. Data was analysed using Stata statistical software. We used descriptive statistics and summarised data as counts (n) and frequencies (%) for categorical variables, and as medians with interquartile ranges for numerical variables.

For analysis of selective outcome reporting (i.e., reporting outcomes in a publication that differ from those planned in the trial protocol) we had four assumptions: 1) There may be a difference in selective outcome reporting when a trial is registered prospectively compared with retrospectively registered trials. 2) There may be selective outcome reporting for trials that had multiple recruitment centres compared to those with single recruitment centres. To test our assumptions, we planned paired binary data test, which was relevant for examining

For peer review only - http://bmjopen.bmj.com/site/about/guidelines.xhtml

trial registration status (prospective or retrospective), our independent variable, and selective outcome reporting in publication "yes or no", our dichotomous variable. To perform a Chi-squared test we conducted expected frequency summary statistics to see if the test was appropriate. A Chi-squared test was possible for our comparison between single country and multi-country trials and for other comparisons, expected counts of most cells were less than five, so we proceeded to conduct a Fisher's exact test to test our assumptions. For time to publication, we used the, Kaplan Meier survival analysis to determine time from trial completion to publication for the overall trials that had publication and for single country versus multi-country trials. When performing time to publication analysis, we concentrated on the occurrence of publishing as some of the trials were ongoing while some trials hadn't started where they will give a future end date, therefore we could not censor. We also performed a Log-rank test to determine difference in time to publication between single country and multi-country trials.

RESULTS

We found 440 trials from the WHO-ICTRP (20 January 2019) and the Russian trials registry (30 March 2019). After eligibility assessment, 187 trials were excluded because they were duplicates of trials registered in more than one primary registry, or the purpose of a trial was not TB treatment, or they were a different study design such as observational study. In total, 253 trials were included in the analysis.

Baseline characteristics

Recruitment centres

When exploring recruitment centres, we found that 30% (77) of the trials were multi-country trials, 22% (55) took place in China, 21% (53) in India, 13% (34) in South Africa, 9% (23) in Russia and 4% (11) in Brazil. Recruitment centres were identified as "single country single-centre" for 46% (117), "multi-country multi-centre" for 30% (77) and "single country multi-centre" for 24% (59) of the trials respectively. Supplementary results are presented in appendix 2.

Registration status

For peer review only - http://bmjopen.bmj.com/site/about/guidelines.xhtml

Sixty-four percent (163) of the trials were registered prospectively, 28% (69) retrospectively and 8% (21) did not report status at registration of the trial. When exploring registration status at country level, we found that prospective registration was 86% for multi-country, 79% for South Africa, 69% for China, 51% for Indian, 36% for Brazil and 4% for Russia. When we compared multi-country vs single country trials, we found that multi-country trials more often registered prospectively compared to individual country trials (86% vs 55%, p =0.00) (Table 1).

Table 1. Registration status of multi-country trials versus single country trials

Registration status	Multiple country trials		Single country trials		Total		P value
	%	n	%	N	%	n	
Not reported	0	0	12	21	8	21	0,00
Prospective	86	66	55	97	64	163	
Retrospective	14	11	33	58	27	69	
Total	100	77	100	176	100	253	

Sample size

The sample sizes of trials ranged from 0 to 1500 000 with a median sample size of 200 (IQR: 92 to 500) where one trial reported zero as a sample size. At country level, trial sample sizes ranged from 20 to 6400 per trial with a median of 145 (IQR 50 - 546) for Brazil; from 40 to 400 participants per trial with a median of 100 (IQR 70 - 170) for Russia; from 0 to 4000 with a median of 175 (IQR 110 - 327) for India; from 16 to 4176 with a median of 300 (IQR 100 - 500) for China; and from 20 to 1500 000 with a median of 394 (IQR 155 - 1155) for multi-country trials.

Age of participants

The majority of the trials 73% (183) recruited adult participants, 6% (15) trials recruited adolescents and adults, 5% (13) trials included all ages, 4% (10) trials included children and adolescents, and less than 1% (1) included children or adolescents only. Eleven percent (30) of the trials did not report ages of participants. Out of the 183 trials that included adult

For peer review only - http://bmjopen.bmj.com/site/about/guidelines.xhtml

For peer review only

participants only 30% (54) of the trials were those conducted in multiple countries, 24% from China, 22% (41) from India, 18% (32) from South Africa, 6% (10) from Brazil and 1% (2) from Russia. In the 30 trials that did not report ages of participants, 67% (20) were from Russia. Two trials that recruited children only and adolescents only, respectively, were multi-country trials.

Type of treatment

Categories of treatment

Trials evaluated different treatment, including 81% (207) studying drug treatment, 9% (22) studying behavioural treatment, 5% (12) nutritional supplementation, 3% (7) evaluating Direct Observed Therapy (DOT), almost 2% (4) evaluating surgical treatment and less than 1% (1) assessing rehabilitation for TB management. For the 207 trials evaluating the impact of drug treatment, 35% (73) were multi-country trials, 20% (41) conducted in China, 18% (37) conducted in India, 13% (28) from South Africa, 11% (22) from Russia and 3% (6) trials from Brazil. Drug treatment was further sub-divided into the type of drug treatment comprising a) new combination treatment; b) new drugs; c) new treatment durations; and d) new dosages as shown in Table 2. When we compared single country and multi-country trials, we found that drug treatment trials accounted for 76% of single country trials and for 95% of multi-country trials (p=0.015)

Table 2. Number of trials per type of drug treatment

**Type of TB drug treatment**	**n**	**%**
New combination	70	34
New drug	59	28
New treatment duration	45	22
New dosage	33	16
Total	207	100

For peer review only - http://bmjopen.bmj.com/site/about/guidelines.xhtml

We further analysed the drug treatment trials according to the phase of the trial and found that most were phase 2 (47), followed by phase 3 (41), and phase 4 (20), However, most of the trials did not report their trial phase (68). The remaining 31 trials were similarly distributed in other phases of trials.

Type of TB studied

Most of the included trials evaluated drug-sensitive TB treatment, 79% (201), followed by trials evaluating drug-resistant TB treatment, 21% (52). For the 201 trials studying drug-sensitive TB, 29% (58) were conducted in multiple countries, 23% (47) in India, 22% (44) in China, 12% (25) in South Africa, 8% (16) in Russia, 5% (11) in Brazil. In the 52 trials studying drug resistant TB, 46% (24) trials conducted in multiple countries, 21% (11) in China, 13% (7) in Russia, 12% (6) in South Africa, and 8% (4) in India.

Co-morbidities and TB management

We found that 19% (47) of included trials evaluated TB treatment in participants with co-morbidities. These included HIV/AIDS (42 trials), diabetes mellitus (three trials) and other multi-morbidities (two trials). The two trials that studied TB treatment in participants with multi-morbidities were conducted in India, while the three trials that studied TB treatment in participants with diabetes were conducted in China (2) and India (1). Fifty percent (21) of trials that evaluated TB treatment in participants with HIV/AIDS were multi-countries trials, 17% (7) were from India, another 17% (7) from South Africa, 5% (2) from China and the other 5% (2) from Brazil. Rheumatoid arthritis was studied as a comorbidity in one trial from China.

Funding sources and Ethics approval

Funding sources

When exploring the funding sources of included trials we found that 25% (64) trials were funded by local organisations, 23% (58) were funded by international organisations, 17% (44) by the pharmaceutical industry, 9% (22) by universities, 6% (15) of the trials claimed to be self- funded and 5% (14) were funded by hospitals. We also found that 8% (19) had multiple funding sources while 7% (17) did not report funding sources (Figure 1).

Funding at the country level in Table 3 shows that majority of the multi-country trials were funded by international organisations (36) and industry (16) while for each of the trials

For peer review only - http://bmjopen.bmj.com/site/about/guidelines.xhtml

funding was mostly by local and international organisation except for Russia where 17 out of 23 was funded by industry. More single country trials compared to multi-country trials had local funding (31% vs 12%,) and multi-country trials had more international funding than single country trials (47% vs 13%) p=0.00.

Table 3. Funding sources at country level

**Funding sources**	**Brazil n (%)**	**Russia n (%)**	**India n (%)**	**China n (%)**	**South Africa n (%)**	**Multi-countries n (%)**	**Total number n**
International organisation	1 (9)	2 (9)	7 (13)	2 (4)	10 (25)	36 (47)	58 (23)
Local organisation	5 (46)	3 (13)	27 (50)	15 (27)	5 (15)	9 (12)	64 (25)
Self-funding	0 (0)	0 (0)	4 (7)	8 (14)	2 (6)	1 (1)	15 (6)
Hospital	1 (9)	1 (4)	0 (0)	11 (20)	0 (0)	1 (1)	14 (5)
Industry	0 (0)	17 (74)	5 (9)	1 (2)	5 (15)	16 (21)	44 (17)
University	3 (27)	0 (0)	5 (9)	5 (9)	5 (15)	4 (5)	22 (9)
Multiple funding	0 (0)	0 (0)	4 (7)	2 (4)	6 (18)	7 (9)	19 (8)
Unclear	1 (9)	0 (0)	1 (2)	11 (20)	1 (3)	3 (4)	17 (7)
Total number	11 (100)	23 (100)	53 (100)	55 (100)	34 (100)	77 (100)	253 (100)

Ethics approval

When exploring whether ethical approval was obtained and where it was obtained from, we found that 50% (125) of the trials had obtained ethics approval locally, 4% (11) of the trials obtained approval only from international ethics review boards, 8% (20) of the trials obtained

For peer review only - http://bmjopen.bmj.com/site/about/guidelines.xhtml

For peer review only

ethics approval from both local and international ethics review boards, while 38% (97) of the trials did not report ethics approval details.

Trial registration linked to publication

We found that 40% (101/ 253 trials) were published and indexed in PubMed. At country level we found that of the 101 published trials, 43% (43) of the publications were from multi-country trials, 23% (23) from India, 16% (16) from South Africa, 13% (13) from China, 4% (4) from Brazil and 1% (2) from Russia.

Selective outcome reporting

There was selective outcome reporting in 31 of 101 published trials as they reported a different primary outcome in the publication to that pre-specified in the trial registry record (table 4). Seventy trials of 101 reported a primary outcome in the publication that matched the outcome pre-specified in the trial registry record. We compared selective outcome reporting in prospectively registered trials versus in retrospectively registered trials and found that results were similar (30% vs 29%, p=0.318). When exploring the data across the BRICS countries we found that the multi-country trials had 19% of selective reporting versus single country trials with 40% of selective reporting (p=0.029).

Table 4. Selective reporting for primary outcomes per country

Primary outcome same as ICTRP	Brazil n (%)	Russia n (%)	India n (%)	China n (%)	South Africa n (%)	Multi-countries n (%)	Total No. (%)
No	2 (50)	2 (100)	8 (35)	6 (46)	5 (31)	8 (20)	31 (30)
Yes	2 (50)	0 (0)	15 (65)	7 (54)	11 (69)	35 (81)	70 (70)
Total	4 (100)	2 (100)	23 (100)	13 (100)	16 (100)	43 (100)	101 (100)

Time to publication

Overall, the median time to publication from the anticipated completion of a trial according to the listed end date in the trial registry record was 26 months (range 0 to 67 month). Multi-

For peer review only - http://bmjopen.bmj.com/site/about/guidelines.xhtml

country trials publish within a median of 23 months (IQR 12-34) which is similar to single-country trials 26 months (IQR 15-37) (p = 0.3117, Log-rank test)

DISCUSSION

Among the 253 included trials, we found that most trials (77) registered in primary registries were those with recruitment centres in multiple countries, followed by in China (55) and India (53). Of the BRICS countries, China and India were listed as countries with the highest burden of drug sensitive TB and two of three countries with the highest burden in MDR-TB [3]. Results from this study show that these two countries account for the majority of the trials studying drug sensitive TB and China with the second most trials studying drug resistant TB following multi-countries trials. Our results also found that many of the BRICS TB trials registered in primary registries are registered prospectively with a statistically significant difference between trial locations where multi-country trials are more likely to be registered prospectively as compared to single country trials. This supports similar studies that have shown an increase in the number of prospectively registered trials [25]. Prospective registration of trials is recommended by the ICJME guidelines to reduce selective outcome reporting and publication bias in an event that a trial that has negative results [20].

When exploring the ages of participants, we found that the majority (73%) of the trials studied adult participants only. While there are a few trials that studied all ages, only one trial studied children from birth to 12 years and one trial studying adolescents 13-17 years of age. This re-iterates the substantial gap in TB treatment research done in children and adolescents where risk factor related research has shown that children are the population most susceptible to transmission of TB and MDR-TB [18]. Children have remained poorly represented in clinical research due to, at times, challenging ethical and regulatory process of involving minors in clinical trials. This was recognised in 2009 prior to the launch PACTR child strategy which advocated for clinical research to be done in children to increase knowledge base of treatment where children are concerned [31].

Eighty one percent of the trials in the BRICS countries studied dug treatment trials, this likely indicates the efforts of these countries to find ways to treat and eradicate TB disease. According to research, there is evidence of effective TB treatment that has been widely

For peer review only - http://bmjopen.bmj.com/site/about/guidelines.xhtml

adopted [2], however trials in these countries are still focused on treatment trials for new drugs approaches, but not enough emphasis on implementation such as behavioural or social aspects of TB including adherence. This is especially important to ensure that where there is availability of effective treatment for TB the efforts are maximised to reduce TB epidemic. Our results also showed that majority of single country trials were funded by local organisations whereas multi-country trials were likely to be funded by international funding, these results were statistically significant. Results also showed that majority of the trials received local ethics approvals with majority of the PIs situated within the BRICS countries. This evidence supports research statement that suggest TB studies in the BRICS region tend to be funded and ran within the countries of recruitment [21]. However, what is concerning is that 38% of the trials did not report having received ethics approval for conducting a trial, questions may arise about the credibility of these trials.

When exploring selective outcome reporting in publications we found that 31 of 101 publications had selective outcome reporting. Results comparing selective outcome reporting by trial location showed that trials conducted in single countries were more likely to have selective outcome reporting than multi-country trials, these results were statistically significant. The results indicate that trials conducted in single countries which are most likely single centre trials are prone to selective outcome reporting. Although the majority of published trials in our small sample have results aligned with their planned outcomes, 30% do not, which constitutes a substantial number and high level of selective outcome reporting. There was no statistically significant difference in selective reporting when comparing prospective and retrospective registration.

In a single centre trial, sample sizes can be small and not enough to dilute differences that may influence outcomes as compared to multicentre trials which may lead to changes in outcome preferences [31]. This may point to the value in investing in larger trials that can be conducted in multicentre to improve outcomes reporting and generalisability of findings.

Lastly, for trials with publications, we explored the time from the presumed end-date found in the registry to publication. The WHO and ICJME recommends publication within 12 months of trial completion [19-20]. Time to publication is on average 26 months and there is no significant difference between various factors such as the country of the trial, or whether a

For peer review only - http://bmjopen.bmj.com/site/about/guidelines.xhtml

For peer review only

single country trial which on average published at 26 months compared to multi-countries trials which on average publishes at 23 months.

CONCLUSION

TB treatment trial activity in BRICS countries, documented by our study, showed that the majority of registered trials were collaborative prospectively registered multi-country trials followed by country level nationally funded and implemented trials in China, India, South Africa and Russia. However, trials in children and adolescents were almost missing from these high-burden countries. The trials are still mostly drug-treatment oriented, despite the recognised gap in research on TB treatment implementation. Time to publication from trial end-date was longer than 2 years, this lag time does not meet current WHO and ICMJE requirements to ensure research results reach the public and impact health policy timely. One third of the published trials had evidence of selective outcome reporting which raises concern and emphasizes the need for researchers to update their trial information regularly once a trial registered to increase transparency, integrity and credibility of their research. Our mapping of TB treatment trials revealed the research gaps, which may guide policymakers, researchers and funders regarding the areas for focus to enhance TB treatment research. This is particularly true in the current situation in BRICS countries where despite having effective treatment to manage TB to date, TB cases remain on the rise affecting many people.

ETHICS

This study was exempted from ethical consideration since the data collected is publicly available and at no point was confidential information from human participants used.

FUNDING

Grant awarded: Not Applicable

Other funding: This work was supported by the South African Medical Research Council (through salaries for LIM, DEN, and TK).

CONTRIBUTORS

LIM led the development of the study, wrote the first draft of the protocol and paper, coordinated and integrated comments from co-authors. All authors will be guarantor of the

For peer review only - http://bmjopen.bmj.com/site/about/guidelines.xhtml

paper. LOM was involved in screening, data extraction and data analysis; DN and LEZ were involved in the screening, data extraction and translation of data from Russian to English; MM, and EJ were involved in data analysis and provided support in the write up of the data analysis and results section. DEN and TK critically provided supervision and mentorship to LIM, together with DN and LEZ critically revised successive drafts of the manuscript, provided important intellectual input. All authors approved the final manuscript prior to submission.

ACKNOWLEDGMENTS

This work was supported by Cochrane South Africa staff at South African Medical Research Council, Tamara Kredo, Elizabeth Pienaar, Duduzile Ndwandwe, and Ameer Hohfeld. We also wish to thank Mikateko Mazinu and Esme Jordan for data analysis support from the South African Medical Research Council Biostats unit.

PATIENT AND PUBLIC INVOLVEMENT

There was no patient or public involvement in the conducting of this study.

COMPETING INTERESTS

At the time of this work, LIM, DEN, EP and TK were all part of the administrative team coordinating the Pan African Clinical Trials Registry (www.pactr.mrc.ac.za) and part of development of a new database for the South African National Clinical Trials registry. DN and LEZ declare no competing interest.

DATA AVAILABILITY STATEMENT

Data will be made available through request; we will provide aggregated data in excel sheet.

For peer review only

REFERENCES

1. Floyd K, Fitzpatrick C, Pantoja A, et al. Domestic and donor financing for tuberculosis care and control in low-income and middle-income countries: an analysis of trends, 2002–11, and requirements to meet 2015 targets. *Lancet Glob Health*. 2013 Aug 1;1(2):e105-15.
2. Nafade V, Nash M, Huddart S, Pande T, et al. A bibliometric analysis of tuberculosis research, 2007–2016. *PloS one*. 2018 Jun 25;13(6):e0199706.
3. Creswell J, Sahu S, Sachdeva KS, et al. Tuberculosis in BRICS: challenges and opportunities for leadership within the post-2015 agenda. *Bulletin of the World Health Organization*. 2014 Jun;92(6):459-60.
4. United Nations Sustainable Development. (2019). WHO calls for urgent action to end TB - United Nations Sustainable Development. [online] Available at: https://www.un.org/sustainabledevelopment/blog/2018/09/who-calls-for-urgent-action-to-end-tb/ [Accessed 14 Aug. 2019].
5. Lienhardt C, Lönnroth K, Menzies D, et al. Translational research for tuberculosis elimination: priorities, challenges, and actions. *PLoS Med*. 2016 Mar 2;13(3):e1001965
6. Lienhardt C, Espinal M, Pai M, Maher D, et al. What research is needed to stop TB? Introducing the TB Research Movement. *PLoS Med*. 2011 Nov 29;8(11):e1001135.
7. Bai J, Li W, Huang YM, et al. Bibliometric study of research and development for neglected diseases in the BRICS. *Infect Dis Poverty*. 2016 Dec;5(1):89.
8. Viergever RF, Terry RF, Karam G. Use of data from registered clinical trials to identify gaps in health research and development. *Bulletin of the World Health Organization*. 2013;91:416-25C.
9. Zeeneldin AA, Taha FM. The Egyptian clinical trials' registry profile: analysis of three trial registries (International Clinical Trials Registry Platform, Pan-African Clinical Trials Registry and clinicaltrials. gov). *J Adv Res*. 2016 Jan 1;7(1):37-45.

For peer review only

For peer review only - http://bmjopen.bmj.com/site/about/guidelines.xhtml

10. Forum on Drug Discovery, Development. The Global Crisis of Drug-Resistant Tuberculosis and Leadership of China and the BRICS: Challenges and Opportunities: Summary of a Joint Workshop by the Institute of Medicine and the Institute of Microbiology, *Chinese Academy of Sciences.*2014.

11. Vera-Badillo FE, Napoleone M, Krzyzanowska MK, Alibhai SM, Chan AW, Ocana A, Seruga B, Templeton AJ, Amir E, Tannock IF. Bias in reporting of randomised clinical trials in oncology. *European Journal of Cancer*. 2016 Jul 1;61:29-35.

12. Zarin DA, Keselman A. Registering a clinical trial in ClinicalTrials. gov. *Chest.* 2007 Mar 1;131(3):909-12.

13. Zarin DA, Ide NC, Tse T, et al. Issues in the registration of clinical trials. *Jama.* 2007 May 16;297(19):2112-20.

14. Dal-Ré R. Improving transparency of clinical trials. *Trends Pharmacol Sci*. 2015 Jun 1;36(6):323-5.

15. Bonnett LJ, Davies GR. Quality of outcome reporting in phase II studies in pulmonary tuberculosis. Trials. 2015 Dec;16(1):518.

16. "What Does All Trials Registered And Reported Mean?". Alltrials, 2018, http://www.alltrials.net/find-out-more/all-trials/. Accessed 12 Sept 2018.

17. Bortolini, MAT. "Registering a clinical trial." (2017): 803-804

18. Hunter KE, Seidler AL, Askie LM. Prospective registration trends, reasons for retrospective registration and mechanisms to increase prospective registration compliance: descriptive analysis and survey. *BMJ open*. 2018 Mar 1;8(3):e019983.

19. Consolidated Standards for Reporting Trials (CONSORT). 2010. http://www.consort-statement.org/. Accessed 23, 2019.

20. De Angelis C, Drazen JM, Frizelle FA, et al. Clinical trial registration: a statement from the International Committee of Medical Journal Editors. *Lancet* 2004;364:911–2.

21. Pai M. Time for high-burden countries to lead the tuberculosis research agenda. 2016

For peer review only

For peer review only - http://bmjopen.bmj.com/site/about/guidelines.xhtml

22. Proehl JA, Alexander S, Manton A. Integrity and Transparency in Reporting Clinical Trials. J Emerg Nurs. 2017 Mar 1;43(2):96-7.

23. Nithyashri J, Kulanthaivel G. Classification of human age based on Neural Network using FG-NET Aging database and Wavelets. In2012 Fourth International Conference on Advanced Computing (ICoAC) 2012 Dec 13 (pp. 1-5). IEEE.

24. Jones CW, Keil LG, Holland WC, et al. Comparison of registered and published outcomes in randomized controlled trials: a systematic review. *BMC Med*. 2015;13:282

25. Jones PM, Chow JT, Arango MF, et al. Comparison of registered and reported outcomes in randomized clinical trials published in anesthesiology journals. *Anesthesia & Analgesia*. 2017 Oct 1;125(4):1292-300.

26. Scott A, Rucklidge JJ, Mulder RT. Is mandatory prospective trial registration working to prevent publication of unregistered trials and selective outcome reporting? An observational study of five psychiatry journals that mandate prospective clinical trial registration. *PloS one*. 2015 Aug 19;10(8):e0133718.

27. Tse T, Fain KM, Zarin DA. How to avoid common problems when using Clinicaltrials. gov in research: 10 issues to consider. *BMJ*. 2018 May 25;361:k1452.

28. Jones CW, Safferman MR, Adams AC, et al. Discrepancies between ClinicalTrials. gov recruitment status and actual trial status: a cross-sectional analysis. *BMJ open*. 2017 Oct 1;7(10):e017719.

29. Askie LM, 2011. Australian New Zealand Clinical Trials Registry: history and growth. *Journal of evidence-based medicine*, *4*(3), pp.185-187.

30. Abrams AL, Opiyo N, Cotton M, et al. Supporting registration of child-focused clinical trials in Africa: The Child Strategy project. *SAMJ.* 2011. 101(11), pp.804-804.

31. Faber J and Fonseca LM. How sample size influences research outcomes. *Dental press journal of orthodontics*, 2014. *19*(4), pp.27-29.

For peer review only - http://bmjopen.bmj.com/site/about/guidelines.xhtml

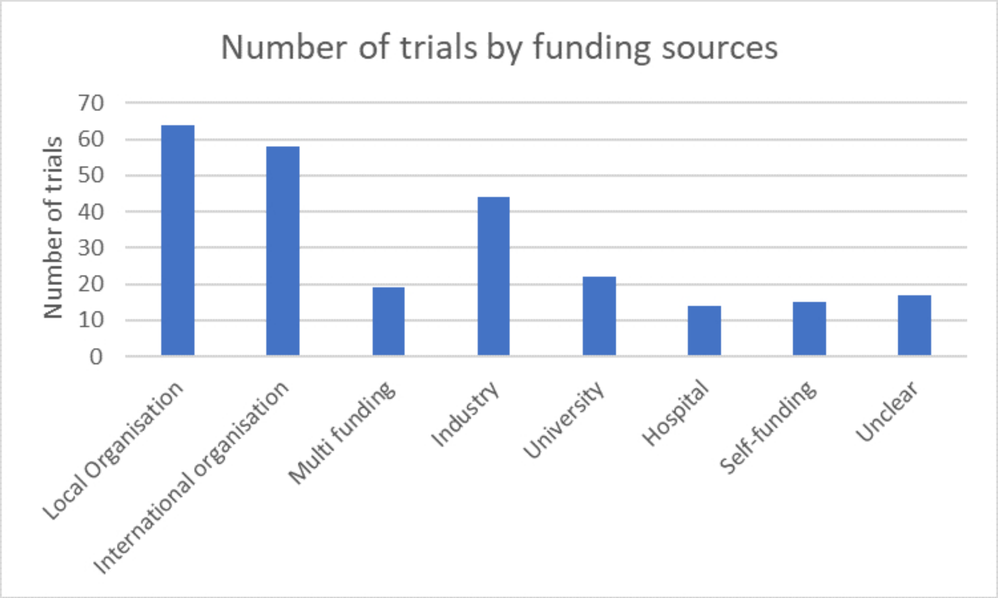

Fig 1. Funding sources

323x194mm (59 x 59 DPI)

Appendix 1

Search terms

“isoniazid”, “rifampicin”, “pyrazinamide”, “ethambutol”, “streptomycin”, “rifabutin”, “kanamycin”, “amikacin”, “capreomycin”, “levofloxacin”, “moxifloxacin”, “ofloxacin”, “para-aminosalicylic acid”, “cycloserine”, “terizidone”, “ethionamide”, “protionamide”, “clofazimine”, “linezolid”, “amoxicillin/clavulanate”, “thioacetazone”, “imipenem/cilastatin”, “high-dose isoniazid”, “clarithromycin”, “bedaquiline”, “delamanid”, “meropenem”.

Coding of variables

Type of intervention
Drug therapy
Behavioral
Dots
Supplementation
Rehabilitation
Surgery

Age of participants	
Children	birth to 12 months
	1 to 5 years
	6 to 12 years
Adolescents only (<18)	13 to 17
Adults only	18 and over
Children and Adolescents	
Adolescents and adults	
All ages	
Not Reported	

For peer review only

Ethics
Local
International
Local and International
Not reported

Funding sources
Local Organisation
International organisation
Multiple funding
Industry
University
Hospital
Self funding
Unclear

Co-morbidities
HIV/AIDS
Diabetes
Rheumatoid disease
Multi-morbidity
Not applicable

Type of drug treatment
New drug
New combination
New treatment duration

For peer review only

New dosagw

For peer review only

Planned, ongoing and completed tuberculosis treatment trials in Brazil, Russia, India, China, and South Africa: A 2019 cross-sectional descriptive analysis

SECTION A

	Primary Outcome					
	No	Not Applicable	Unclear	Yes	Total	P value
	n(%)	n(%)	n(%)	n(%)	No.	
Registrationstat						
Not reported	1 (5)	20 (95)	0 (0)	0 (0)	21	
Prospective	16 (10)	97 (60)	4 (3)	46 (28)	163	0.01*
Retrospective	7 (10)	35 (51)	3 (4)	24 (35)	69	
Total	24 (10)	152 (60)	7 (3)	70 (28)	253	
**CountriesSinglecountryMultin**						
Multi-national	6 (8)	36 (47)	1 (1)	34 (44)	77	
Sinngle country	18 (10)	116 (66)	6 (3)	36 (21)	176	0.002
Total	24 (10)	152 (60)	7 (3)	70 (28)	253	
**Funding source grouped**						
Local_Organisation	9 (14)	31 (48)	1 (2)	23 (36)	64	
International_organisation	4 (7)	29 (50)	4 (7)	21 (36)	58	
multifunding	3 (16)	10 (53)	0 (0)	6 (32)	19	
Industry	4 (9)	32 (73)	0 (0)	8 (18)	44	
University	2 (9)	15 (68)	1 (5)	4 (18)	22	-
Hospital	1 (7)	10 (71)	1 (7)	2 (14)	14	
Self_funding	1 (7)	11 (73)	0 (0)	3 (20)	15	
Not_reported	0 (0)	14 (82)	0 (0)	3 (18)	17	
Total	24 (10)	152 (60)	7 (3)	70 (28)	253	
**Trial Location Country**						
Brazil	1 (9)	7 (64)	1 (9)	2 (18)	11	
China	6 (11)	42 (76)	0 (0)	7 (13)	55	
India	8 (15)	30 (57)	0 (0)	15 (28)	53	
Multiple countries	7 (9)	34 (44)	1 (1)	35 (46)	77	-
Russia	1 (4)	21 (91)	1 (4)	0 (0)	23	
South Africa	1 (3)	18 (53)	4 (12)	11 (32)	34	
Total	24 (10)	152 (60)	7 (3)	70 (28)	253	

*If not reported excluded, p-value = 0.544

Fisher exact test is used to calculate the p value. There is significant difference between registration status and primary outcome. However, if we exclude "Not reported" category, no significance difference is observed between the two groups. Funding source and trial location has small cell number, thus no p value provided.

For peer review only

Planned, ongoing and completed tuberculosis treatment trials in Brazil, Russia, India, China, and South Africa: A 2019 cross-sectional descriptive analysis

Trial contries grouped

RecrutimentSinglecentreMulti	Multiple countries		Single countries		Total		P value
	%	n	%	n	%	n	
Multicentre	56	75	44	59	100	134	0.00
Single centre	1.7	2	98.3	117	100	119	
Total	30.4	77	69.6	176	100	253	

TriallocationCountry

Registrationstat	Brazil		China		India		Multiple countries		Russia		South Africa		Total	
	%	n	%	n	%	n	%	n	%	n	%	n	%	n
Not reported	0	0	0	0	0	0	0	0	91.3	21	0	0	8.3	21
Prospective	36.4	4	69.1	38	50.9	27	85.7	66	4.3	1	79.4	27	64.4	163
Retrospective	63.6	7	30.9	17	49.1	26	14.3	11	4.3	1	20.6	7	27.3	69
Total	100	11	100	55	100	53	100	77	100	23	100	34	100	253

Trial contries grouped

For peer review only

Planned, ongoing and completed tuberculosis treatment trials in Brazil, Russia, India, China, and South Africa: A 2019 cross-sectional descriptive analysis

Registrationstat	Multiple countries		Single countries		Total		P value
	%	n	%	n	%	n	
Not reported	0	0	11.9	21	8.3	21	0.00
Prospective	85.7	66	55.1	97	64.4	163	
Retrospective	14.3	11	33	58	27.3	69	
Total	100	77	100	176	100	253	

TriallocationCountry

Recruitmentstat	Brazil		China		India		Multiple countries		Russia		South Africa		Total	
	%	No.	%	No.	%	No.	%	No.	%	No.	%	No.	%	No.
Complete	0	0	0	0	0	0	1.3	1	4.3	1	0	0	0.8	2
Not recruiting	81.8	9	65.5	36	77.4	41	70.1	54	4.3	1	76.5	26	66	167
Not reported	0	0	0	0	0	0	2.6	2	91.3	21	0	0	9.1	23
Recruiting	18.2	2	34.5	19	22.6	12	24.7	19	0	0	23.5	8	23.7	60
Terminated	0	0	0	0	0	0	1.3	1	0	0	0	0	0.4	1
Total	100	11	100	55	100	53	100	77	100	23	100	34	100	253

Trial contries grouped

Recruitmentstat	Multiple countries	Single countries	Total	P value

For peer review only

Planned, ongoing and completed tuberculosis treatment trials in Brazil, Russia, India, China, and South Africa: A 2019 cross-sectional descriptive analysis

	%	No.	%	No.	%	No.	
Complete	1.3	1	0.6	1	0.8	2	
Not recruiting	70.1	54	64.2	113	66	167	
Not reported	2.6	2	11.9	21	9.1	23	0.039
Recruiting	24.7	19	23.3	41	23.7	60	
Terminated	1.3	1	0	0	0.4	1	
Total	100	77	100	176	100	253	

Trial contries grouped

Typeofintervention	Multiple countries		Single countries		Total		P value
	%	No.	%	No.	%	No.	
Behavioral	3.9	3	10.8	19	8.7	22	
DOTs	0	0	4	7	2.8	7	
Drug therapy	94.8	73	76.1	134	81.8	207	
Rehabilitation	0	0	0.6	1	0.4	1	0.015
Supplementation	1.3	1	6.3	11	4.7	12	
Surgery	0	0	2.3	4	1.6	4	
Total	100	77	100	176	100	253	

TriallocationCountry

Funding source grouped	Brazil	China	India	Multiple countries	Russia	South Africa	Total

For peer review only

Planned, ongoing and completed tuberculosis treatment trials in Brazil, Russia, India, China, and South Africa: A 2019 cross-sectional descriptive analysis

	%	No.	%	No.	%	No.	%	No.	%	No.	%	No.	%	No.
Local_Organisation	45.5	5	27.3	15	50.9	27	11.7	9	13	3	14.7	5	25.3	64
International_organisation	9.1	1	3.6	2	13.2	7	46.8	36	8.7	2	29.4	10	22.9	58
multifunding	0	0	3.6	2	7.5	4	9.1	7	0	0	17.6	6	7.5	19
Industry	0	0	1.8	1	9.4	5	20.8	16	73.9	17	14.7	5	17.4	44
University	27.3	3	9.1	5	9.4	5	5.2	4	0	0	14.7	5	8.7	22
Hospital	9.1	1	20	11	0	0	1.3	1	4.3	1	0	0	5.5	14
Self_funding	0	0	14.5	8	7.5	4	1.3	1	0	0	5.9	2	5.9	15
Other	9.1	1	20	11	1.9	1	3.9	3	0	0	2.9	1	6.7	17
Total	100	11	100	55	100	53	100	77	100	23	100	34	100	253

	Trial contries grouped						
Funding source grouped	Multiple countries		Single countries		Total		P value*
	%	No.	%	No.	%	No.	
Local_Organisation	11.7	9	31.3	55	25.3	64	
International_organisation	46.8	36	12.5	22	22.9	58	
multifunding	9.1	7	6.8	12	7.5	19	
Industry	20.8	16	15.9	28	17.4	44	0.00
University	5.2	4	10.2	18	8.7	22	
Hospital	1.3	1	7.4	13	5.5	14	
Self_funding	1.3	1	8	14	5.9	15	
Other	3.9	3	8	14	6.7	17	

*chi 2 used

For peer review only

Planned, ongoing and completed tuberculosis treatment trials in Brazil, Russia, India, China, and South Africa: A 2019 cross-sectional descriptive analysis

Total	100	77	100	176	100	253	

TriallocationCountry

Primary Outcome	Brazil		China		India		Multiple countries		Russia		South Africa		Total	
	%	No.	%	No.	%	No.	%	No.	%	No.	%	No.	%	No.
Yes	50	2	53.8	7	65.2	15	81.4	35	0	0	68.8	11	69.3	70
No_Unclear	50	2	46.2	6	34.8	8	18.6	8	100	2	31.3	5	30.7	31
Total	100	4	100	13	100	23	100	43	100	2	100	16	100	101

Trial contries grouped

Primary Outcome	Multiple countries		Single countries		Total		P value
	%	No.	%	No.	%	No.	
Yes	81.4	35	60.3	35	69.3	70	0.029
No_Unclear	18.6	8	39.7	23	30.7	31	
Total	100	43	100	58	100	101	

For peer review only

Planned, ongoing and completed tuberculosis treatment trials in Brazil, Russia, India, China, and South Africa: A 2019 cross-sectional descriptive analysis

SECTION B: Survival analysis.

In this section, we exclude 9 observations that published studies before end trial date.

This graph shows the overall Kaplan-Meier survival estimate, with a median publication time of 25 months.

For peer review only

Planned, ongoing and completed tuberculosis treatment trials in Brazil, Russia, India, China, and South Africa: A 2019 cross-sectional descriptive analysis

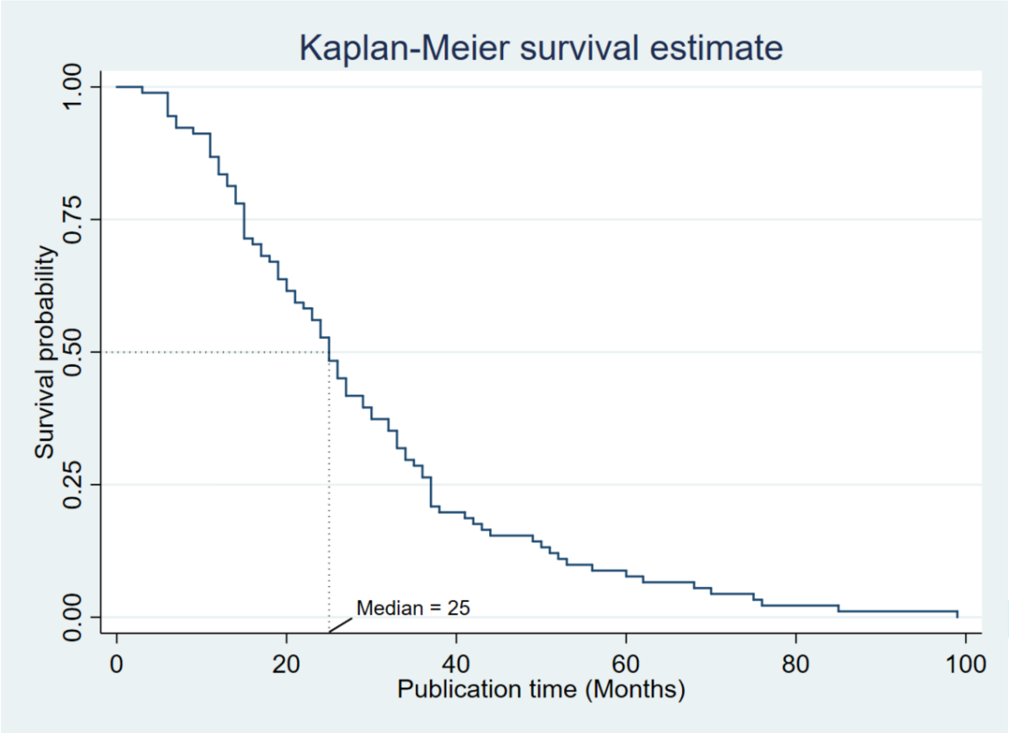

Planned, ongoing and completed tuberculosis treatment trials in Brazil, Russia, India, China, and South Africa: A 2019 cross-sectional descriptive analysis

This graph shows the overall Kaplan-Meier survival estimate for single country with a median publication time of 26 months.

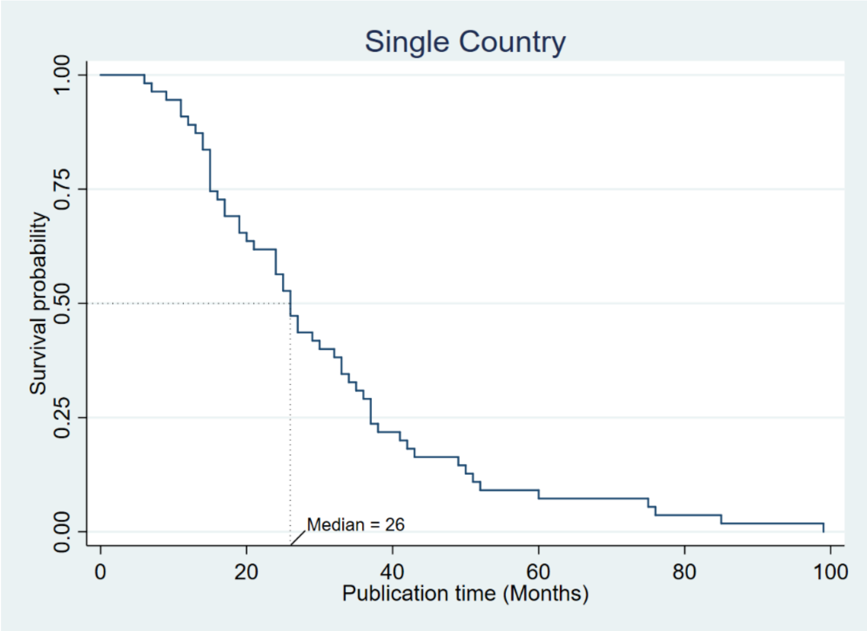

This graph shows the overall Kaplan-Meier survival estimate for multinational studies with a median publication time of 23 months. This shows that multinational studies takes shorter time to publish as compared with single country study.

Planned, ongoing and completed tuberculosis treatment trials in Brazil, Russia, India, China, and South Africa: A 2019 cross-sectional descriptive analysis

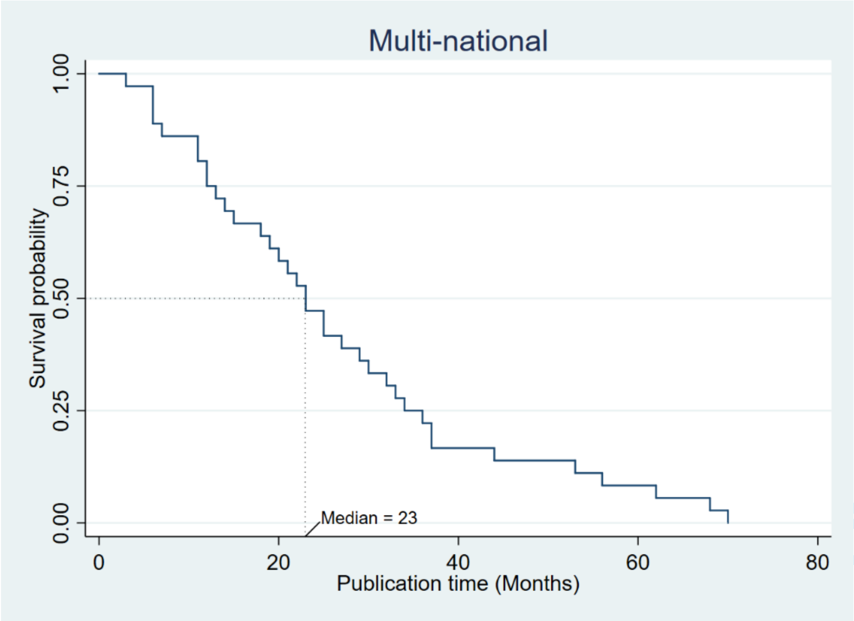

This graph shows the overall Kaplan-Meier survival estimate for India trial location with a median publication time of 29 months.

For peer review only

Planned, ongoing and completed tuberculosis treatment trials in Brazil, Russia, India, China, and South Africa: A 2019 cross-sectional descriptive analysis

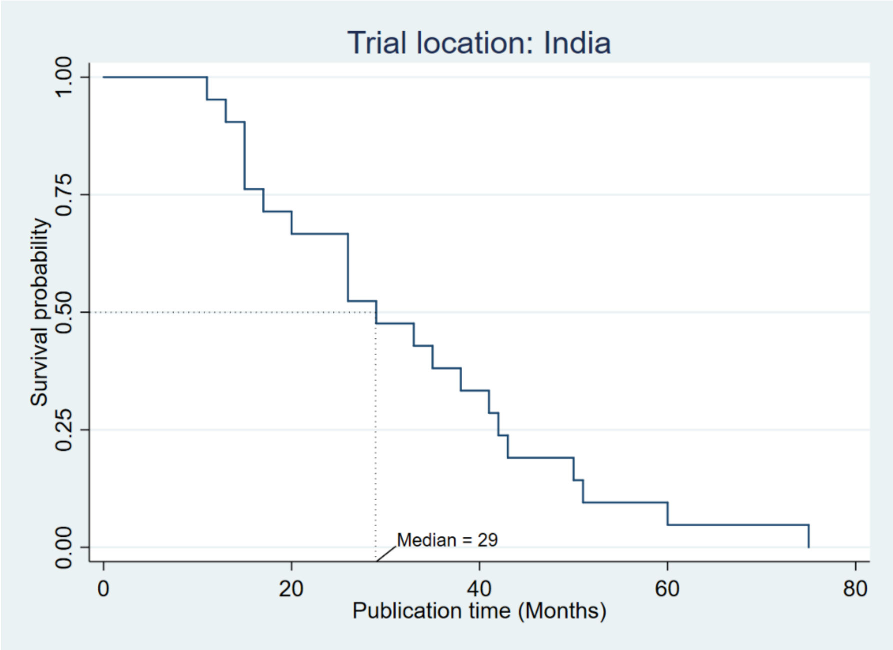

This graph shows the overall Kaplan-Meier survival estimate for China trial location with a median publication time of 25 months.

For peer review only - http://bmjopen.bmj.com/site/about/guidelines.xhtml

Planned, ongoing and completed tuberculosis treatment trials in Brazil, Russia, India, China, and South Africa: A 2019 cross-sectional descriptive analysis

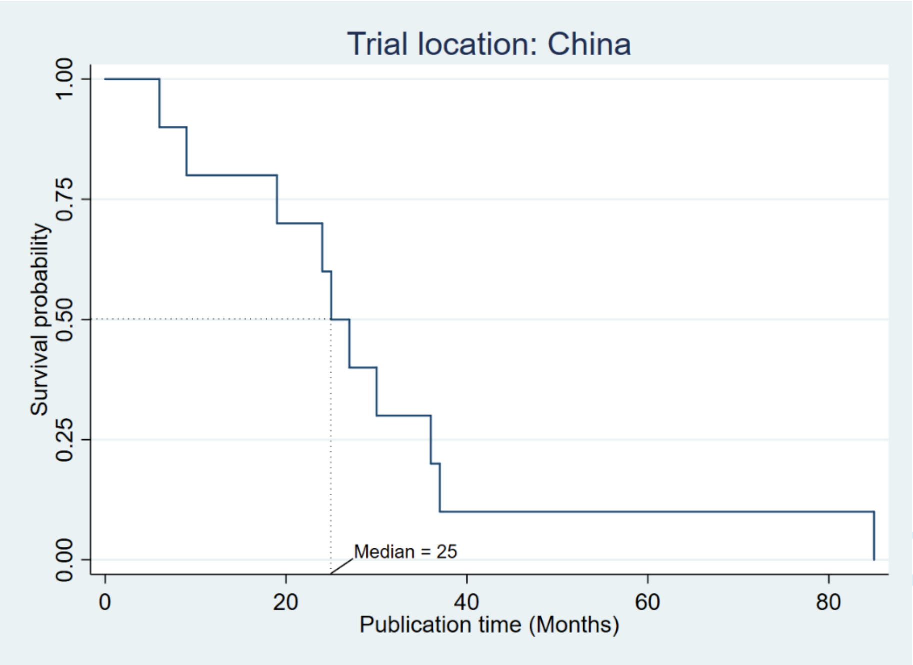

This graph shows the overall Kaplan-Meier survival estimate for Multi-countries trial location with a median publication time of 23 months.

For peer review only - http://bmjopen.bmj.com/site/about/guidelines.xhtml

Planned, ongoing and completed tuberculosis treatment trials in Brazil, Russia, India, China, and South Africa: A 2019 cross-sectional descriptive analysis

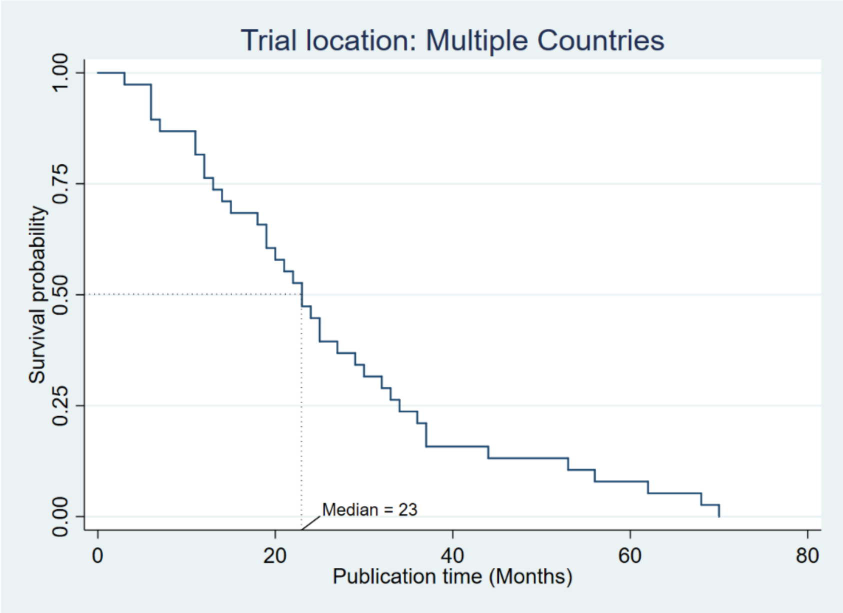

This graph shows the overall Kaplan-Meier survival estimate for combine Brazil, Russia and South Africa trial location with a median publication time of 24 months.

Planned, ongoing and completed tuberculosis treatment trials in Brazil, Russia, India, China, and South Africa: A 2019 cross-sectional descriptive analysis

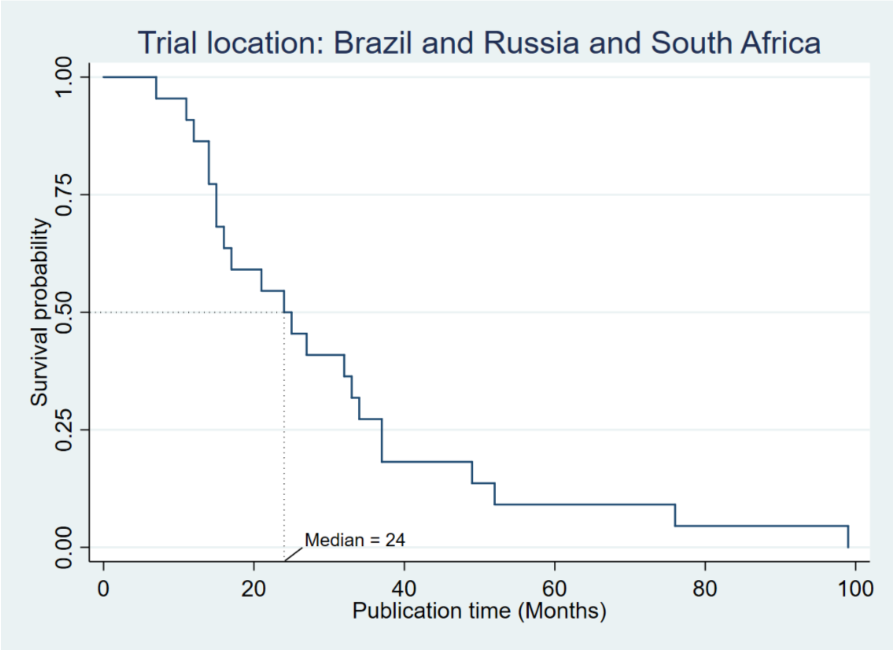

Multi-countries trial location studies takes shorter time to publish as compared to the other trial locations.

For peer review only

Planned, ongoing and completed tuberculosis treatment trials in Brazil, Russia, India, China, and South Africa: A 2019 cross-sectional descriptive analysis

For peer review only

Planned, ongoing and completed tuberculosis treatment trials in Brazil, Russia, India, China, and South Africa: A 2019 cross-sectional descriptive analysis

Journal:	*BMJ Open*
Manuscript ID	bmjopen-2021-057941.R1
Article Type:	Original research
Date Submitted by the Author:	26-Apr-2022
Complete List of Authors:	Mathebula, Lindi; South African Medical Research Council, Cochrane South Africa; Stellenbosch University, Department of Global Health<br>Mapahla, Lovemore; Stellenbosch University, Department of Global Health; South African Medical Research Council, Biostatistics unit<br>Nurkhametova, Dilyara; Russian Medical Academy for Continuing Professional Education of the Ministry of Health<br>Ziganshina, Liliya Eugenevna; Russian Medical Academy for Continuing Professional Education of the Ministry of Health; Kazan Medical University and RUDN University<br>Mazinu, Mikateko; Medical Research Council of South Africa, Biostatistics unit<br>Jordan, Esme; South African Medical Research Council, Biostatistics unit; University of the Western Cape, Statistics and Population Studies Department<br>Ndwandwe, Duduzile; South African Medical Research Council, Cochrane South Africa<br>Kredo, Tamara; South African Medical Research Council, Cochrane South Africa; Stellenbosch University Faculty of Medicine and Health Sciences, Division of Clinical Pharmacology, Department of Medicine,
**Primary Subject Heading**:	Infectious diseases
Secondary Subject Heading:	Public health
Keywords:	Tuberculosis < INFECTIOUS DISEASES, Clinical trials < THERAPEUTICS, Respiratory infections < THORACIC MEDICINE

I, the Submitting Author has the right to grant and does grant on behalf of all authors of the Work (as defined in the below author licence), an exclusive licence and/or a non-exclusive licence for contributions from authors who are: i) UK Crown employees; ii) where BMJ has agreed a CC-BY licence shall apply, and/or iii) in accordance with the terms applicable for US Federal Government officers or employees acting as part of their official duties; on a worldwide, perpetual, irrevocable, royalty-free basis to BMJ Publishing Group Ltd ("BMJ") its licensees and where the relevant Journal is co-owned by BMJ to the co-owners of the Journal, to publish the Work in this journal and any other BMJ products and to exploit all rights, as set out in our licence.

The Submitting Author accepts and understands that any supply made under these terms is made by BMJ to the Submitting Author unless you are acting as an employee on behalf of your employer or a postgraduate student of an affiliated institution which is paying any applicable article publishing charge ("APC") for Open Access articles. Where the Submitting Author wishes to make the Work available on an Open Access basis (and intends to pay the relevant APC), the terms of reuse of such Open Access shall be governed by a Creative Commons licence – details of these licences and which Creative Commons licence will apply to this Work are set out in our licence referred to above.

Other than as permitted in any relevant BMJ Author's Self Archiving Policies, I confirm this Work has not been accepted for publication elsewhere, is not being considered for publication elsewhere and does not duplicate material already published. I confirm all authors consent to publication of this Work and authorise the granting of this licence.

For peer review only

Submission for BMJ Open

Planned, ongoing and completed tuberculosis treatment trials in Brazil, Russia, India, China, and South Africa: A 2019 cross-sectional descriptive analysis

Lindi Mathebula[1,2*], Lovemore Mapahla[2,4], Dilyara Nurkhametova[3], Liliya Eugenevna Ziganshina[3,6], Mikateko Mazinu[4], Esme Jordan[4,5], Duduzile ndwandwe[1], Tamara Kredo[1, 2]

1. Cochrane South Africa, South African Medical Research Council, Tygerberg, Cape Town, South Africa
2. Division of Epidemiology and Biostatistics, Department of Global Health, Stellenbosch University, Cape Town, South Africa
3. Cochrane Russia, Russian Medical Academy for Continuing Professional Education of the Ministry of Health, Moscow, Russian Federation and St Albans House, 57-59 Haymarket, London, United Kingdom
4. Biostatistics unit, South African Medical Research Council, Tygerberg, Cape Town, South Africa
5. Statistics and Population Studies Department, University of the Western Cape
6. Kazan Medical University and RUDN University, Moscow, Russian Federation
7. Division of Clinical Pharmacology, Department of Medicine, Faculty of Medicine and health Sciences, Stellenbosch University, Cape Town, South Africa

* Corresponding author

E-mail: lindi.mathebula@mrc.ac.za

Keywords: *Tuberculosis, Treatment, Trials, BRICS, Primary registries*

For peer review only - http://bmjopen.bmj.com/site/about/guidelines.xhtml

Submission for BMJ Open

ABSTRACT

Tuberculosis (TB) remains a deadly challenge globally and Brazil, Russia, India, China, and South Africa (BRICS) are amongst the countries with the highest TB burden. The objective of this study is to identify and describe ongoing, planned, and completed TB trials conducted in the BRICS countries registered in World Health Organisation International Clinical Trial Registry (WHO-ICTRP); to report selective outcome reporting by comparing primary outcomes in published trials with their pre-specified outcomes in registry records and to evaluate the time to publication.

Methods and analysis: We searched the WHO-ICTRP portal (20 January 2019) and the Russian Federation Registry (30 March 2019) to identify TB trials conducted in BRICS countries. We included only registered clinical trials conducted wholly in BRICS countries or with at least one recruitment centre in one of the BRICS countries that were investigating TB treatment.

Results: The search of the WHO-ICTRP yielded 408 trials and additional 32 trials were identified from the Russian registry. Of those, 253 were included in the analysis. We found that 77 trials were multi-country trials, followed by trials in China (55), India (53), South Africa (34), Russia (23), and Brazil (11). One hundred and sixty-three (163) trials were registered prospectively, 69 retrospectively, and 21 trials had no registration status. Most trials (207) evaluated TB treatment, followed by 29 behaviour change interventions, 13 nutritional supplementation, four surgical treatment and two assessing rehabilitation. Based on ICJME recommendation of publishing 12 months after completion of trial, we found that 156 trials were completed 12 or more months by date and 101 trials had publications. Thirty-one of the 101 trials with publication had evidence of selective outcome reporting. The median time to publication was 25 months (IQR 15-37) from the time of anticipated end date stated in the registry.

Conclusion: TB trials conducted in BRICS countries are collaborative, mostly drug treatment oriented, potentially affecting policies. Selective outcome reporting remains a problem both for prospectively and retrospectively registered trials, only small fraction of which gets to publication.

For peer review only

For peer review only - http://bmjopen.bmj.com/site/about/guidelines.xhtml

STRENGTHS AND LIMITATIONS OF THIS STUDY

- This study addresses a research gap by describing and analysing planned, ongoing and completed TB treatment trials specific to middle-income countries with high TB burden, Brazil, Russia, India, China and South Africa.
- We conducted an extensive search of relevant clinical trials registries including the WHO-ICTRP and the Russian Trials Registry to ensure that no publicly available trial is missed.
- The selection of trials and data extraction were conducted in duplicate to minimise bias and ensure rigor.
- Since initial search on the 20th of January 2019, there have been 20 more publication from included clinical trials. Studies published after our analysis are unlikely to change the description analysis we have performed.
- As not all trials are registered or submitted to the WHO-ICTRP, some trials may have been missed.

For peer review only

For peer review only - http://bmjopen.bmj.com/site/about/guidelines.xhtml

INTRODUCTION

Tuberculosis (TB) remains one of the most challenging and deadliest infectious diseases in the world ranking as the ninth cause of death worldwide [1-2]. Although TB mortality and incidence have been reported to be decreasing by 3% and 2% respectively, most countries have been lagging behind in meeting the United Nations 2030 Sustainable Development Goal of ending the TB epidemic [2]. Brazil, Russia, India, China, and South Africa, (BRICS) countries, have been reported to have a very high burden of TB, hosting 46% of all global TB cases and 40% of TB related deaths [3]. Furthermore, antimicrobial resistance has led to increasing cases of Multi-Drug Resistant TB (MDR-TB) of which China, India, and Russia account for more than half (56%) of the global burden [3]. In addition to the Sustainable Development Goals launched in 2015 aiming to end TB by 2030; the World Health Organization (WHO) launched a strategy in 2014 called the WHO's End TB strategy, which is endorsed by the World Health Assembly, to eradicate the global TB epidemic by 2035 [3-4].

The WHO's End TB 2035 strategy aims to decrease TB related deaths by at least 95% and an overall 90% reduction in TB incidence to 10 cases/100 000 or fewer with TB-affected households experiencing no costs of TB related care [5]. One of the three pillars of the WHO End TB strategy is research and innovation. While research studies regularly estimate incidence, mortality rates and TB management approaches, new innovations of treatments are often not easily tracked [2]. To track TB research efforts, Lienhardt et al suggested a strategic plan that included mapping research funding involvement to determine where the research funding gaps are [6]. Another component of this plan is a Global TB Research Roadmap, which identified research gaps that should be closed in order to stop TB [6]. A study by Bai et al (2018) aimed to conduct a bibliometric analysis of research and development (R&D) using publications to find out what contributions the BRICS countries make in the area for neglected tropical diseases including TB [7]. Another approach to identify research activity in different areas suggested by Viergever et al is to use data from registered trials in primary clinical trials registries [8].

Mapping clinical trial activity through clinical trial registries provides an insight into where most research has been conducted and what type of research is being done or is planned and ongoing [8-10]. Clinical trials are primary research methods that are undertaken to study effects of new interventions such as treatment [11]. Randomised controlled trials (RCTs) have

For peer review only - http://bmjopen.bmj.com/site/about/guidelines.xhtml

been described as the gold standard study design to inform policy and practice decisions regarding what interventions work better for improving healthcare [12]. However, studies showed that 37% of clinical trials presented in abstract and registered in trial registries never reach publication [12-13]. Furthermore, those that reach publication often omit negative results and deviate from pre-specified outcomes leading to publication bias [12-14]. A systematic review looking at quality of outcome reporting in phase 2 studies of TB treatment showed evidence that these studies often had variations between reported outcomes and trial characteristics [15]. Research has suggested that for trials to be transparent, trialists should register their trials prospectively in a public platform indicating the aim of the study and the outcomes to be measured before the trial begins, to minimise selective outcome reporting (mismatch between registered and reported outcomes) [16].

Primary registries are online platforms that register and make public planned clinical trials and the data from different primary registries is aggregated and also made publicly available in the WHO International Clinical Trial Registry Platform (WHO-ICTRP) [17]. This then creates the necessary public accountability mechanism to ensure that planned trials and their results cannot be hidden regardless of their outcomes, which reduces the temptation to not publish results that are negative [18]. Prospective registration of trial in a primary registry means registration of a trial before it starts and is now widely recognized as a key strategy to increase research transparency. Prospective registration is supported by the Consolidated Standards of Reporting Trials (CONSORT) Statement and The International Committee of Medical Journals Editors (ICMJE), which includes many of the world's leading journals [18-20]. Retrospective registration of a trial means a trial that is registered after the first participant has been enrolled into the trial and is not a recommended practice as it diminishes research integrity according to the ICJME and CONSORT statement [18-20].

BRICS countries have become major contributors to clinical research worldwide. Funding for research in the TB field and the number of publications has been seen to increase on a yearly basis having almost doubled in the past decade [21]. Research shows there is a likelihood of omitting or deviating from outcomes or measurement of outcomes when results are not favorable in trials including TB trials [14-15]. This may lead to overestimation of the efficacy of interventions and lead to adverse outcomes when implemented into policy. Proehl et al (2018) has indicated the need for transparency in clinical trials from trial registration to

For peer review only - http://bmjopen.bmj.com/site/about/guidelines.xhtml

publication in order to effect a positive change in policy [22]. In light of this, the objective of this study is to identify and describe ongoing, planned, and completed TB trials conducted in the BRICS countries registered in WHO-ICTRP and report any selective outcome reporting by identifying registered trials that have been published to compare reported outcomes with those pre-specified in the trial registration. Mapping TB treatment trials will assist researchers to identify potential research gaps, sponsors and funders to identify funding gaps, patients to identify available treatments, policy makers to identify evidence-based interventions to end TB epidemic.

OBJECTIVES

The main objective of the study is to identify and describe ongoing, planned, and completed TB trials conducted in the BRICS countries registered in WHO-ICTRP and report any selective outcome reporting by identifying registered trials that have been published to compare reported outcomes with those pre-specified in the trial registration.

Hypothesis

The hypothesis of this study is that there is a difference between outcomes specified trial publications and those pre-specified in the trial registration.

For peer review only

For peer review only - http://bmjopen.bmj.com/site/about/guidelines.xhtml

MATERIALS AND METHODS

Study design

This is a cross-sectional study, in which we describe and analyse data from registered trials studying TB treatment in BRICS countries found on WHOs clinical trial registry platform

Study Sample

We aimed to include all registered trials planned for conduct in BRICS countries from inception of the WHO-ICTRP data repository in 2005 to the date of search. We searched the WHO-ICTRP portal on 20 January 2019 to identify TB trials conducted in BRICS countries using the following search terms: “tuberculosis” AND “Treatment” OR “Therapy” OR “Management” AND “Country-name” i.e each of the BRICS countries. Two researchers Lindi Mathebula (LIM) and Lovemore Mapahla (LOM) independently screened all records of the search and selected trials to be included. The Russian Federation does not have a primary registry that submits trials data to the WHO-ICTRP platform. Therefore, two researchers Dilyara Nurkhametova (DN) and Liliya Eugenevna Ziganshina (LEZ) from Cochrane Russia (based at Russian Medical Academy for Continuing Professional Education, the Russian Federation), searched the Russian trial registry, named the State Registry of Medicines (GRLS, grls.rosminzdrav.ru) (30 March 2019). This registry contains trial information on all clinical trials officially approved by the Federal Ministry of health. Since the Russian registry does not allow for an advanced search with Boolean operators, we searched for trials using the term “tuberculosis” followed by a series of separate searches for each of the following antituberculosis medicines (search terms available in appendix 1).

We included only registered clinical trials regardless of status of the trial (i.e. ongoing, planned, or completed) conducted wholly in BRICS countries or with at least one recruitment centre in one of the BRICS countries that were investigating interventions for the treatment of TB. Trials were either randomised controlled trials, controlled clinical trials including cluster trials, or multiple-arm trials. Upon retrieval of the search output, we removed studies that were observational from the sample and excluded duplicates and trials that were not eligible. WHO-ICTRP considers the first trial record registered to take precedence for trials registered in more than one primary register, however we considered the trial record with the latest

For peer review only - http://bmjopen.bmj.com/site/about/guidelines.xhtml

date and excluded the other records to ensure that we have a full record of the changes that were made from earlier trial registration.

Patient and Public Involvement

We had no public or patient involvement in the conduct of this study.

After selection of the trials into the study, two researchers (LIM, LOM) independently used trial identification numbers to search for publications in PubMed and Cochrane Library. DN and LEZ searched the Russian electronic database of research publications eLIBRARY.RU using trial identification numbers and antituberculous medicines names. Publications were further identified in the trial record under the field "URL to publication" which is one of the 24 data items that is collected by WHO-ICTRP primary registries.

Data extraction

Trial records

We downloaded data from the WHO-ICTRP and imported into Microsoft Excel™ with the following data items: registry name, trial identification, initial application date, start date of trial, end date of trial, date of registration, registration status (retrospective/prospective), principal investigator, source of funding, type of treatment, primary and secondary outcomes, inclusion/exclusion criteria, age of participants, and sample size amongst other baseline characteristics. Data from the Russian trial registry was manually entered in the excel format under the same data items. Two researchers (LIM, LOM) independently reviewed data from the WHO-ICTRP manually and extracted further data points from individual records of included trials, similarly two researchers (LEZ, DN) from Russia independently and manually reviewed data from the Russian trial registry and extracted further data points from individual records. The researchers translated records that were in Russian language to English before sending data in Excel format.

Publication extraction

Two researchers (LIM, LOM) independently extracted data from all published studies of the included trials in Excel for comparison. The following data items were extracted from the publication: title of the study, publication date, trial identification listed (yes/no), study design, age of participants, sample size, primary outcomes. We combined the data extracted

For peer review only

by the two researchers from South Africa and two researchers from the Russian Federation for cleaning and coding. For selective outcome reporting we compared what was reported in the trial record as a primary outcome including the type of outcome and time points for the measurement of the outcome, to what was reported in the publication as a primary outcome. We used "yes" if the authors reported the same outcome and "no" if they reported a different primary outcome in the publication. We coded the data using variables in categories that unified the data (Appendix 1).

Data Analysis

Once entered data was reviewed and cleaned to ensure that no data was missing, the completed and cleaned data set was imported into the StataCorp. 2015 for statistical analysis. All data was stored on the South African Medical Research Council network and backed up on a daily basis to ensure no loss of original data. Data was analysed using Stata statistical software. We used descriptive statistics and summarised data as counts (n) and frequencies (%) for categorical variables, and as medians with interquartile ranges for numerical variables.

For analysis of selective outcome reporting (i.e., reporting outcomes in a publication that differ from those planned in the trial protocol) we had two assumptions: 1) There may be a difference in selective outcome reporting when a trial is registered prospectively compared with retrospectively registered trials. 2) There may be selective outcome reporting for trials that had multiple recruitment centres compared to those with single recruitment centres. To test our assumptions, we planned paired binary data test, which was relevant for examining trial registration status (prospective or retrospective), our independent variable, and selective outcome reporting in publication "yes or no", our dichotomous variable. To perform a Chi-squared test we conducted expected frequency summary statistics to see if the test was appropriate. A Chi-squared test was possible for our comparison between single country and multi-country trials and for other comparisons, expected counts of most cells were less than five, so we proceeded to conduct a Fisher's exact test to test our assumptions. For time to publication, we used the, Kaplan Meier survival analysis to determine time from trial completion to publication for the overall trials that had publication and for single country versus multi-country trials. When performing time to publication analysis, we concentrated on the occurrence of publishing as some of the trials were ongoing while some trials hadn't started where they will give a future end date, therefore we could not censor. We also

For peer review only - http://bmjopen.bmj.com/site/about/guidelines.xhtml

performed a Log-rank test to determine difference in time to publication between single country and multi-country trials.

RESULTS

We found 440 trials from the WHO-ICTRP (20 January 2019) and the Russian trials registry (30 March 2019) (figure 1). After eligibility assessment, 187 trials were excluded because they were duplicates of trials registered in more than one primary registry, or the purpose of a trial was not TB treatment, or they were a different study design such as observational study. In total, 253 trials were included in the analysis.

Baseline characteristics

Recruitment centres

When exploring recruitment centres, we found that 30% (77) of the trials were multi-country trials, 22% (55) took place in China, 21% (53) in India, 13% (34) in South Africa, 9% (23) in Russia and 4% (11) in Brazil. Recruitment centres were identified as "single country single-centre" for 46% (117), "multi-country multi-centre" for 30% (77) and "single country multi-centre" for 24% (59) of the trials respectively.

Registration status

Sixty-four percent (163) of the trials were registered prospectively, 28% (69) retrospectively and 8% (21) did not report status at registration of the trial. When exploring registration status at country level, we found that prospective registration was 86% for multi-country, 79% for South Africa, 69% for China, 51% for Indian, 36% for Brazil and 4% for Russia. When we compared multi-country vs single country trials, we found that multi-country trials more often registered prospectively compared to individual country trials (86% vs 55%, p =0.00) (Table 1).

For peer review only - http://bmjopen.bmj.com/site/about/guidelines.xhtml

Table 1. Registration status of multi-country trials versus single country trials

Registration status	Multiple country trials		Single country trials		Total		P value
	%	n	%	N	%	n	
Not reported	0	0	12	21	8	21	
Prospective	86	66	55	97	64	163	0,00
Retrospective	14	11	33	58	27	69	
Total	100	77	100	176	100	253	

Sample size

The sample sizes of trials ranged from 0 to 1500 000 with a median sample size of 200 (IQR: 92 to 500) where one trial as identified from trial registry reported zero as a sample size. At country level, trial sample sizes ranged from 20 to 6400 per trial with a median of 145 (IQR 50 - 546) for Brazil; from 40 to 400 participants per trial with a median of 100 (IQR 70 - 170) for Russia; from 0 to 4000 with a median of 175 (IQR 110 - 327) for India; from 16 to 4176 with a median of 300 (IQR 100 - 500) for China; and from 20 to 1500 000 with a median of 394 (IQR 155 - 1155) for multi-country trials.

Age of participants

The majority of the trials 73% (183) recruited adult participants, 6% (15) trials recruited adolescents and adults, 5% (13) trials included all ages, 4% (10) trials included children and adolescents, and less than 1% (1) included children or adolescents only. Eleven percent (30) of the trials did not report ages of participants. Out of the 183 trials that included adult participants only 30% (54) of the trials were those conducted in multiple countries, 24% from China, 22% (41) from India, 18% (32) from South Africa, 6% (10) from Brazil and 1% (2) from Russia. In the 30 trials that did not report ages of participants, 67% (20) were from Russia. Two trials that recruited children only and adolescents only, respectively, were multi-country trials.

For peer review only - http://bmjopen.bmj.com/site/about/guidelines.xhtml

For peer review only

Type of treatment

Categories of treatment

Trials evaluated different treatment, including 81% (207) studying drug treatment, 9% (22) studying behavioural treatment, 5% (12) nutritional supplementation, 3% (7) evaluating Direct Observed Therapy (DOT), almost 2% (4) evaluating surgical treatment and less than 1% (1) assessing rehabilitation for TB management. For the 207 trials evaluating the impact of drug treatment, 35% (73) were multi-country trials, 20% (41) conducted in China, 18% (37) conducted in India, 13% (28) from South Africa, 11% (22) from Russia and 3% (6) trials from Brazil. Drug treatment was further sub-divided into the type of drug treatment comprising a) new combination treatment; b) new drugs; c) new treatment durations; and d) new dosages as shown in Table 2. When we compared single country and multi-country trials, we found that drug treatment trials accounted for 76% of single country trials and for 95% of multi-country trials (p=0.015)

Table 2. Number of trials per type of drug treatment

Type of TB drug treatment	n	%
New combination	70	34
New drug	59	28
New treatment duration	45	22
New dosage	33	16
Total	207	100

We further analysed the drug treatment trials according to the phase of the trial and found that most were phase 2 (47), followed by phase 3 (41), and phase 4 (20), However, most of the trials did not report their trial phase (68). The remaining 31 trials were similarly distributed in other phases of trials such as phase 1 (11), phase 1/2 (9), and phase 2/3 (11).

For peer review only - http://bmjopen.bmj.com/site/about/guidelines.xhtml

Type of TB studied

Most of the included trials evaluated drug-sensitive TB treatment, 79% (201), followed by trials evaluating drug-resistant TB treatment, 21% (52). For the 201 trials studying drug-sensitive TB, 29% (58) were conducted in multiple countries, 23% (47) in India, 22% (44) in China, 12% (25) in South Africa, 8% (16) in Russia, 5% (11) in Brazil. In the 52 trials studying drug resistant TB, 46% (24) trials conducted in multiple countries, 21% (11) in China, 13% (7) in Russia, 12% (6) in South Africa, and 8% (4) in India.

Co-morbidities and TB management

We found that 19% (47) of included trials evaluated TB treatment in participants with co-morbidities. These included HIV/AIDS (42 trials), diabetes mellitus (three trials) and other multi-morbidities (two trials). The two trials that studied TB treatment in participants with multi-morbidities were conducted in India, while the three trials that studied TB treatment in participants with diabetes were conducted in China (2) and India (1). Fifty percent (21) of trials that evaluated TB treatment in participants with HIV/AIDS were multi-countries trials, 17% (7) were from India, another 17% (7) from South Africa, 5% (2) from China and the other 5% (2) from Brazil. Rheumatoid arthritis was studied as a comorbidity in one trial from China.

Funding sources and Ethics approval

Funding sources

When exploring the funding sources of included trials, we found that 25% (64) trials were funded by local organisations, 23% (58) were funded by international organisations, 17% (44) by the pharmaceutical industry, 9% (22) by universities, 6% (15) of the trials claimed to be self- funded and 5% (14) were funded by hospitals. We also found that 8% (19) had multiple funding sources while 7% (17) did not report funding sources (Figure 2).

Funding at the country level in Table 3 shows that majority of the multi-country trials were funded by international organisations (36) and industry (16) while for each of the trials funding was mostly by local and international organisation except for Russia where 17 out of

For peer review only - http://bmjopen.bmj.com/site/about/guidelines.xhtml

23 was funded by industry. More single country trials compared to multi-country trials had local funding (31% vs 12%,) and multi-country trials had more international funding than single country trials (47% vs 13%) p=0.00 (Appendix 2).

Table 3. Funding sources at country level

Funding sources	Brazil n (%)	Russia n (%)	India n (%)	China n (%)	South Africa n (%)	Multi-countries n (%)	Total number n (%)
International organisation	1 (9)	2 (9)	7 (13)	2 (4)	10 (25)	36 (47)	58 (23)
Local organisation	5 (46)	3 (13)	27 (50)	15 (27)	5 (15)	9 (12)	64 (25)
Self-funding	0 (0)	0 (0)	4 (7)	8 (14)	2 (6)	1 (1)	15 (6)
Hospital	1 (9)	1 (4)	0 (0)	11 (20)	0 (0)	1 (1)	14 (5)
Industry	0 (0)	17 (74)	5 (9)	1 (2)	5 (15)	16 (21)	44 (17)
University	3 (27)	0 (0)	5 (9)	5 (9)	5 (15)	4 (5)	22 (9)
Multiple funding	0 (0)	0 (0)	4 (7)	2 (4)	6 (18)	7 (9)	19 (8)
Unclear	1 (9)	0 (0)	1 (2)	11 (20)	1 (3)	3 (4)	17 (7)
Total number	11 (100)	23 (100)	53 (100)	55 (100)	34 (100)	77 (100)	253 (100)

Ethics approval

When exploring whether ethical approval was obtained and where it was obtained from, we found that 50% (125) of the trials had obtained ethics approval locally, 4% (11) of the trials obtained approval only from international ethics review boards, 8% (20) of the trials obtained ethics approval from both local and international ethics review boards, while 38% (97) of the trials did not report ethics approval details.

Trial registration linked to publication

For peer review only - http://bmjopen.bmj.com/site/about/guidelines.xhtml

For peer review only

When focusing of publications linked to trial registration, we found that 156 trials that were completed 12 months or more by date were eligible to have a publication. Of these, we found that 65% (101/ 156 trials) were published and indexed in PubMed. At country level we found that of the 101 published trials, 43% (43) of the publications were from multi-country trials, 23% (23) from India, 16% (16) from South Africa, 13% (13) from China, 4% (4) from Brazil and 1% (2) from Russia.

Selective outcome reporting

There was selective outcome reporting in 31 of 101 published trials as they reported a different primary outcome in the publication to that pre-specified in the trial registry record (table 4). Seventy trials of 101 reported a primary outcome in the publication that matched the outcome pre-specified in the trial registry record. We compared selective outcome reporting in prospectively registered trials versus in retrospectively registered trials and found that results were similar (30% vs 29%, p=0.318). When exploring the data across the BRICS countries we found that the multi-country trials had 19% of selective reporting versus single country trials with 40% of selective reporting (p=0.029).

Table 4. Selective reporting for primary outcomes per country

Primary outcome same as ICTRP	Brazil n (%)	Russia n (%)	India n (%)	China n (%)	South Africa n (%)	Multi-countries n (%)	Total No. (%)
No	2 (50)	2 (100)	8 (35)	6 (46)	5 (31)	8 (20)	31 (30)
Yes	2 (50)	0 (0)	15 (65)	7 (54)	11 (69)	35 (81)	70 (70)
Total	4 (100)	2 (100)	23 (100)	13 (100)	16 (100)	43 (100)	101 (100)

Time to publication

Overall, the median time to publication from the anticipated completion of a trial according to the listed end date in the trial registry record was 26 months (range 0 to 67 month). Multi-country trials publish within a median of 23 months (IQR 12-34) which is similar to single-country trials 26 months (IQR 15-37) (p = 0.3117, Log-rank test).

For peer review only - http://bmjopen.bmj.com/site/about/guidelines.xhtml

DISCUSSION

Among the 253 included trials, we found that most trials (77) registered in primary registries were those with recruitment centres in multiple countries, followed by in China (55) and India (53). Of the BRICS countries, China and India were listed as countries with the highest burden of drug sensitive TB and two of three countries with the highest burden in MDR-TB [3]. Results from this study show that these two countries account for the majority of the trials studying drug sensitive TB and China with the second most trials studying drug resistant TB following multi-countries trials. Our results also found that many of the BRICS TB trials registered in primary registries are registered prospectively with a statistically significant difference between trial locations where multi-country trials are more likely to be registered prospectively as compared to single country trials. This supports similar studies that have shown an increase in the number of prospectively registered trials [23]. Prospective registration of trials is recommended by the ICJME guidelines to reduce selective outcome reporting and publication bias in an event that a trial that has negative results [20].

Even with an increase in prospective registration, we note that researchers, once they register their studies before trial begins, that records are rarely updated. We found a trial that had a sample of zero which was a planned trial and not yet recruiting, however at the time of our analysis, that trial and many others were not updated despite their dates of enrolment and completion listed. If trial records are not updated with amendments, , it leaves room for end users to question the transparency of the research being conducted and make assumptions about the trials [24].

When exploring the ages of participants, we found that the majority (73%) of the trials studied adult participants only. While there are a few trials that studied all ages, only one trial studied children from birth to 12 years and one trial studying adolescents 13-17 years of age. This re-iterates the substantial gap in TB treatment research done in children and adolescents where risk factor related research has shown that children are the population most susceptible to

For peer review only - http://bmjopen.bmj.com/site/about/guidelines.xhtml

transmission of TB and MDR-TB [18]. Children have remained poorly represented in clinical research due to, at times, challenging ethical and regulatory process of involving minors in clinical trials. This was recognised in 2009 prior to the launch PACTR child strategy which advocated for clinical research to be done in children to increase knowledge base of treatment where children are concerned [25].

Eighty one percent of the trials in the BRICS countries studied dug treatment trials, this likely indicates the efforts of these countries to find ways to treat and eradicate TB disease. According to research, there is evidence of effective TB treatment that has been widely adopted [2], however trials in these countries are still focused on treatment trials for new drugs approaches, but not enough emphasis on implementation such as behavioural or social aspects of TB including adherence. This is especially important to ensure that where there is availability of effective treatment for TB the efforts are maximised to reduce TB epidemic. It would be of interest to explore in future research whether the research, trials and testing infrastructure that has been developed for COVID-19 pandemic, especially diagnostics such as PCR might have transferrable value to the TB domain and could offer capacity to support TB and MDR TB research, trials and treatment in the future.

Our results showed that the majority of single country trials were funded by local organisations whereas multi-country trials were likely to be funded by international funding, these results were statistically significant. Results also showed that majority of the trials received local ethics approvals with majority of the PIs situated within the BRICS countries. This evidence supports research statement that suggest TB studies in the BRICS region tend to be funded and ran within the countries of recruitment [21]. However, what is concerning is that 38% of the trials did not report having received ethics approval for conducting a trial, questions may arise about the credibility of these trials.

When exploring selective outcome reporting in publications we found that 31 of 101 publications had selective outcome reporting. Results comparing selective outcome reporting by trial location showed that trials conducted in single countries were more likely to have selective outcome reporting than multi-country trials, these results were statistically significant. The results indicate that trials conducted in single countries which are most likely single centre trials are prone to selective outcome reporting. Although the majority of published trials in our small sample have results aligned with their planned outcomes, 30%

For peer review only - http://bmjopen.bmj.com/site/about/guidelines.xhtml

do not, which constitutes a substantial number and high level of selective outcome reporting. There was no statistically significant difference in selective reporting when comparing prospective and retrospective registration.

In a single centre trial, sample sizes can be small and not enough to dilute differences that may influence outcomes as compared to multicentre trials which may lead to changes in outcome preferences [25]. This may point to the value in investing in larger trials that can be conducted in multicentre to improve outcomes reporting and generalisability of findings.

Lastly, for trials with publications, we explored the time from the presumed end-date found in the registry to publication. The WHO and ICJME recommends publication within 12 months of trial completion [19-20]. We found that the majority of the trials (65%) completed 12 months or more by date had publication. Time to publication is on average 26 months and there is no significant difference between various factors such as the country of the trial, or whether a single country trial which on average published at 26 months compared to multi-countries trials which on average publishes at 23 months. Delays in study completion may be one of the reasons for delayed publication, but as registry records are rarely updated with actual study completion dates, this would require further exploratory research to understand the reasons for delays in depth.

The limitations of this study include that not all trials are registered or submitted to the WHO-ICTRP platform, some trials may have been missed. It is evident that researchers do not always update their trial information including anticipated end date and that may impact correct calculation of the Kaplan Meier, recruitment status may mislead the number of trials that are in fact completed but have not been updated, and there may be deviations from primary outcomes listed that are not updated in the trial registry record. Twenty more publication from included clinical trials since our initial search. Studies published after our analysis are unlikely to change the description analysis we have performed.

CONCLUSION

TB treatment trial activity in BRICS countries, documented by our study, showed that the majority of registered trials were prospectively registered multi-country trials followed by country level nationally funded trials in China, India, South Africa and Russia. However, trials in children and adolescents were almost missing from these high-burden countries. The

For peer review only - http://bmjopen.bmj.com/site/about/guidelines.xhtml

majority of trials are investigating drug-treatment, despite the recognised gap in research on TB treatment implementation. Time to publication from trial end-date was longer than 2 years, this lag time does not meet current WHO and ICMJE requirements to ensure research results reach the public and impact health policy timely. One third of the published trials had evidence of selective outcome reporting which raises concern and emphasizes the need for researchers to update their trial information regularly once a trial registered to increase transparency, integrity and credibility of their research. Our mapping of TB treatment trials revealed the research gaps, which may guide policymakers, researchers and funders regarding the areas for focus to enhance TB treatment research. This is particularly true in the current situation in BRICS countries where despite having effective treatment to manage TB to date, TB cases remain on the rise affecting many people.

ETHICS

This study was exempted from ethical consideration since the data collected is publicly available and at no point was confidential information from human participants used.

FUNDING

Grant awarded: Not Applicable

Other funding: This work was supported by the South African Medical Research Council (through salaries for LIM, DEN, and TK).

CONTRIBUTORS

LIM led the development of the study, wrote the first draft of the protocol and paper, coordinated and integrated comments from co-authors. All authors will be guarantor of the paper. LOM was involved in screening, data extraction and data analysis; DN and LEZ were involved in the screening, data extraction and translation of data from Russian to English; MM, and EJ were involved in data analysis and provided support in the write up of the data analysis and results section. DEN and TK critically provided supervision and mentorship to LIM, together with DN and LEZ critically revised successive drafts of the manuscript, provided important intellectual input. All authors approved the final manuscript prior to submission.

ACKNOWLEDGMENTS

For peer review only - http://bmjopen.bmj.com/site/about/guidelines.xhtml

This work was supported by Cochrane South Africa staff at South African Medical Research Council, Tamara Kredo, Elizabeth Pienaar, Duduzile Ndwandwe, and Ameer Hohfeld. We also wish to thank Mikateko Mazinu and Esme Jordan for data analysis support from the South African Medical Research Council Biostats unit.

COMPETING INTERESTS

At the time of this work, LIM, DEN, EP and TK were all part of the administrative team coordinating the Pan African Clinical Trials Registry (www.pactr.mrc.ac.za) and part of development of a new database for the South African National Clinical Trials registry. DN and LEZ declare no competing interest.

DATA AVAILABILITY STATEMENT

No additional data available.

FIGURE LEGEND

Figures 1 – Prisma flow diagram

Figure 2 – Number of trials by funding sources

For peer review only - http://bmjopen.bmj.com/site/about/guidelines.xhtml

REFERENCES

1. Floyd K, Fitzpatrick C, Pantoja A, et al. Domestic and donor financing for tuberculosis care and control in low-income and middle-income countries: an analysis of trends, 2002–11, and requirements to meet 2015 targets. *Lancet Glob Health*. 2013 Aug 1;1(2):e105-15.

2. Nafade V, Nash M, Huddart S, Pande T, et al. A bibliometric analysis of tuberculosis research, 2007–2016. *PloS one*. 2018 Jun 25;13(6):e0199706.

3. Creswell J, Sahu S, Sachdeva KS, et al. Tuberculosis in BRICS: challenges and opportunities for leadership within the post-2015 agenda. *Bulletin of the World Health Organization*. 2014 Jun;92(6):459-60.

4. United Nations Sustainable Development. (2019). WHO calls for urgent action to end TB - United Nations Sustainable Development. [online] Available at: https://www.un.org/sustainabledevelopment/blog/2018/09/who-calls-for-urgent-action-to-end-tb/ [Accessed 14 Aug. 2019].

5. Lienhardt C, Lönnroth K, Menzies D, et al. Translational research for tuberculosis elimination: priorities, challenges, and actions. *PLoS Med*. 2016 Mar 2;13(3):e1001965

6. Lienhardt C, Espinal M, Pai M, Maher D, et al. What research is needed to stop TB? Introducing the TB Research Movement. *PLoS Med*. 2011 Nov 29;8(11):e1001135.

7. Bai J, Li W, Huang YM, et al. Bibliometric study of research and development for neglected diseases in the BRICS. *Infect Dis Poverty*. 2016 Dec;5(1):89.

8. Viergever RF, Terry RF, Karam G. Use of data from registered clinical trials to identify gaps in health research and development. *Bulletin of the World Health Organization*. 2013;91:416-25C.

9. Zeeneldin AA, Taha FM. The Egyptian clinical trials' registry profile: analysis of three trial registries (International Clinical Trials Registry Platform, Pan-African Clinical Trials Registry and clinicaltrials. gov). *J Adv Res*. 2016 Jan 1;7(1):37-45.

For peer review only

For peer review only - http://bmjopen.bmj.com/site/about/guidelines.xhtml

10. Forum on Drug Discovery, Development. The Global Crisis of Drug-Resistant Tuberculosis and Leadership of China and the BRICS: Challenges and Opportunities: Summary of a Joint Workshop by the Institute of Medicine and the Institute of Microbiology, *Chinese Academy of Sciences.*2014.

11. Vera-Badillo FE, Napoleone M, Krzyzanowska MK, Alibhai SM, Chan AW, Ocana A, Seruga B, Templeton AJ, Amir E, Tannock IF. Bias in reporting of randomised clinical trials in oncology. *European Journal of Cancer*. 2016 Jul 1;61:29-35.

12. Zarin DA, Keselman A. Registering a clinical trial in ClinicalTrials. gov. *Chest.* 2007 Mar 1;131(3):909-12.

13. Zarin DA, Ide NC, Tse T, et al. Issues in the registration of clinical trials. *Jama.* 2007 May 16;297(19):2112-20.

14. Dal-Ré R. Improving transparency of clinical trials. *Trends Pharmacol Sci*. 2015 Jun 1;36(6):323-5.

15. Bonnett LJ, Davies GR. Quality of outcome reporting in phase II studies in pulmonary tuberculosis. Trials. 2015 Dec;16(1):518.

16. "What Does All Trials Registered And Reported Mean?". Alltrials, 2018, http://www.alltrials.net/find-out-more/all-trials/. Accessed 12 Sept 2018.

17. Bortolini, MAT. "Registering a clinical trial." (2017): 803-804

18. Hunter KE, Seidler AL, Askie LM. Prospective registration trends, reasons for retrospective registration and mechanisms to increase prospective registration compliance: descriptive analysis and survey. *BMJ open*. 2018 Mar 1;8(3):e019983.

19. Consolidated Standards for Reporting Trials (CONSORT). 2010. http://www.consort-statement.org/. Accessed 23, 2019.

20. De Angelis C, Drazen JM, Frizelle FA, et al. Clinical trial registration: a statement from the International Committee of Medical Journal Editors. *Lancet* 2004;364:911–2.

21. Pai M. Time for high-burden countries to lead the tuberculosis research agenda. 2016

For peer review only

For peer review only - http://bmjopen.bmj.com/site/about/guidelines.xhtml

580 22. Proehl JA, Alexander S, Manton A. Integrity and Transparency in Reporting Clinical Trials. J Emerg Nurs. 2017 Mar 1;43(2):96-7.

582 23. Jones PM, Chow JT, Arango MF, et al. Comparison of registered and reported outcomes in randomized clinical trials published in anesthesiology journals. *Anesthesia & Analgesia*. 2017 Oct 1;125(4):1292-300.

585 24. Ndwandwe DE, Runeyi S, Pienaar E, et alPractices and trends in clinical trial registration in the Pan African Clinical Trials Registry (PACTR): a descriptive analysis of registration data. *BMJ Open* 2022;12:e057474.

588 25. Faber J and Fonseca LM. How sample size influences research outcomes. *Dental press journal of orthodontics*, 2014. *19*(4), pp.27-29.

590

For peer review only

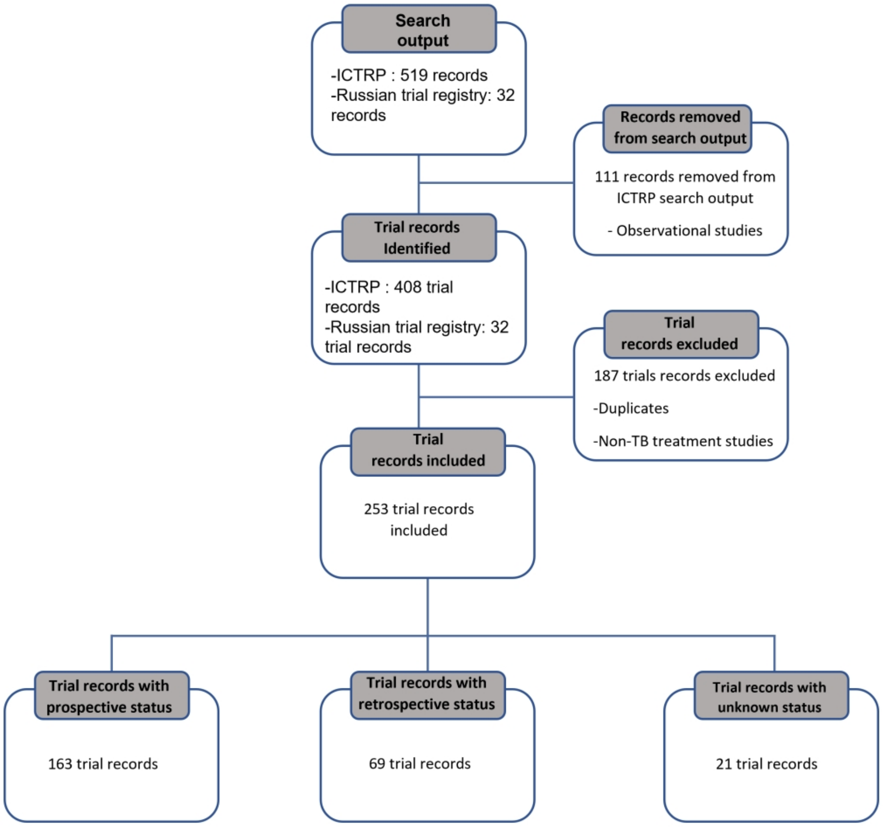

Prisma flow diagram

630x693mm (130 x 130 DPI)

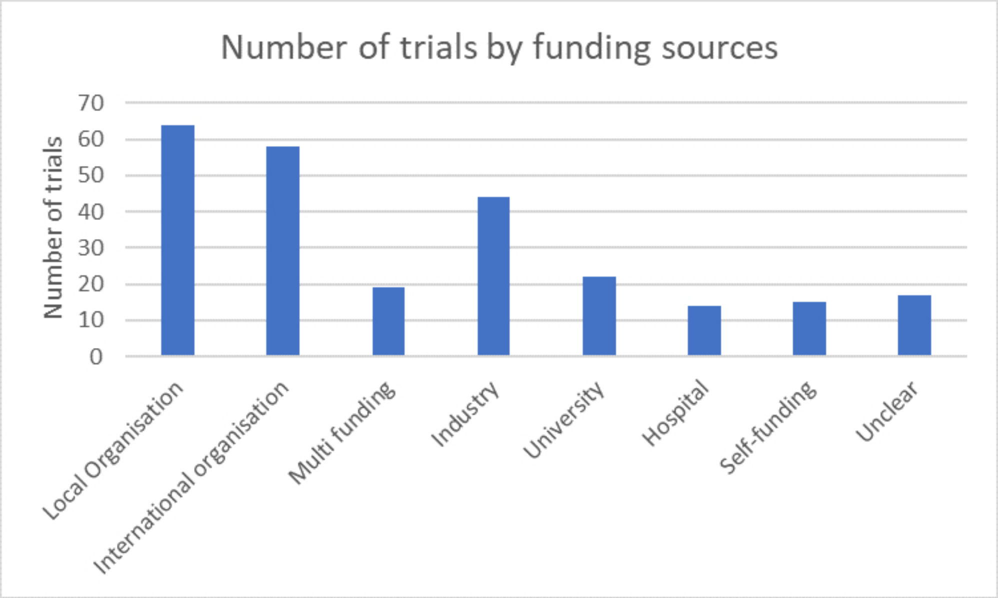

Number of trials by funding sources

323x194mm (59 x 59 DPI)

Appendix 1

Search terms

“isoniazid”, “rifampicin”, “pyrazinamide”, “ethambutol”, “streptomycin”, “rifabutin”, “kanamycin”, “amikacin”, “capreomycin”, “levofloxacin”, “moxifloxacin”, “ofloxacin”, “para-aminosalicylic acid”, “cycloserine”, “terizidone”, “ethionamide”, “protionamide”, “clofazimine”, “linezolid”, “amoxicillin/clavulanate”, “thioacetazone”, “imipenem/cilastatin”, “high-dose isoniazid”, “clarithromycin”, “bedaquiline”, “delamanid”, “meropenem”.

For peer review only

Coding of variables

Type of intervention
Drug therapy
Behavioral
Dots
Supplementation
Rehabilitation
Surgery

Age of participants	
Children	birth to 12 months
	1 to 5 years
	6 to 12 years
Adolescents only (<18)	13 to 17
Adults only	18 and over
Children and Adolescents	
Adolescents and adults	
All ages	
Not Reported	

Ethics
Local
International
Local and International
Not reported

Funding sources
Local Organisation
International organisation
Multiple funding
Industry
University
Hospital
Self funding
Unclear

Co-morbidities
HIV/AIDS
Diabetes
Rheumatoid disease
Multi-morbidity
Not applicable

Type of drug treatment
New drug
New combination
New treatment duration
New dosage

For peer review only

For peer review only

Planned, ongoing and completed tuberculosis treatment trials in Brazil, Russia, India, China, and South Africa: A 2019 cross-sectional descriptive analysis

APPENDIX 2

SECTION A: Basic analysis

Table 1. Two-way table showing primary outcome reporting on trials completed 12 or more months by date versus registration status, recruiting country type, funding sources, and trial location.

	Primary outcome at completion	Primary outcome same for trial and published record	Primary outcome not the same for trial and published record	Total	P value
	**n(%)**	**n(%)**	**n(%)**	**No.**	
**Registration status**					
Not reported	7 (94)	0 (0)	1 (6)	8	0.001
Prospective	41 (38)	46 (43)	20 (19)	107	
Retrospective	7 (17)	24 (59)	10 (24)	41	
**Total**	**55 (35)**	**70 (45)**	**31 (20)**	**156**	
**Recruiting country type**					
Multi-national	42 (50)	35 (41)	8 (9)	85	0.0017
Single country	13 (18)	35 (49)	23 (32)	71	
**Total**	**55 (35)**	**70 (45)**	**31 (20)**	**156**	
**Funding source grouped**					
Local_Organisation	7 (17)	19 (48)	14 (35)	40	-
International_organisation	11 (28)	21 (52)	7 (18)	39	
Multifunding	3 (50)	3 (50)	0 (0)	6	
Industry	11 (39)	12 (43)	5 (18)	28	
University	3 (20)	7 (70)	1 (10)	10	
Hospital	6 (50)	3 (25)	3 (25)	12	
Self_funding	7 (70)	2 (20)	1 (10)	10	
Not_reported	7 (70)	3 (30)	0 (0)	10	
**Total**	**55 (35)**	**70 (45)**	**31 (20)**	**156**	
**Trial Location Country**					
Brazil	3 (60)	1 (20)	1 (20)	5	-
China	7 (64)	2 (13)	2 (13)	11	
India	2 (7)	17 (63)	8 (30)	27	
Multiple countries	22 (58)	10 (26)	6 (16)	38	
Russia	7 (27)	14 (54)	5 (19)	26	
South Africa	14 (29)	26 (53)	9 (18)	49	
**Total**	**55 (35)**	**70 (45)**	**31 (20)**	**156**	

Fisher exact test is used to calculate the p value. There is significant difference between registration status and primary outcome. There is also significance difference observed between recruitment

For peer review only

Planned, ongoing and completed tuberculosis treatment trials in Brazil, Russia, India, China, and South Africa: A 2019 cross-sectional descriptive analysis

country type and primary outcome. Funding source and trial location has small cell number, thus no p value provided.

Table 2. Two-way table comparing recruitment centres beween recruitment country types

Recruitment centres	Multiple countries		Single countries		Total		P value
	%	n	%	n	%	n	0.00
**Multi-centre**	56	77	44	59	100	136	
**Single centre**	0	0	98.3	117	100	119	
**Total**	30.4	77	69.6	176	100	253	

Fisher exact test is used to calculate the p value. There is significant difference when comparing recruitment centres between recruitment country types.

Table 3. Two-way table showing registration status versus trial location countries

Registration status	Brazil		China		India		Multiple countries		Russia		South Africa		Total	
	%	n	%	n	%	n	%	n	%	n	%	n	%	n
**Not reported**	0	0	0	0	0	0	0	0	91.3	21	0	0	8.3	21
**Prospective**	36.4	4	69.1	38	50.9	27	85.7	66	4.3	1	79.4	27	64.4	163
**Retrospective**	63.6	7	30.9	17	49.1	26	14.3	11	4.3	1	20.6	7	27.3	69
**Total**	100	11	100	55	100	53	100	77	100	23	100	34	100	253

Table 3: Two-way table comparing registration status between recruiting country type

Registration status	Multiple countries		Single countries		Total		P value
	%	n	%	n	%	n	0.00
**Not reported**	0	0	11.9	21	8.3	21	
**Prospective**	85.7	66	55.1	97	64.4	163	
**Retrospective**	14.3	11	33	58	27.3	69	
**Total**	100	77	100	176	100	253	

Fisher exact test is used to calculate the p value. There is significant difference when comparing registration status between recruitment country type.

Table 4: Two-way table comparing recruitment status between recruitment country types

Recruitment status	Multiple countries		Single countries		Total		P value
	%	No.	%	No.	%	No.	0.039
**Complete**	1.3	1	0.6	1	0.8	2	
**Not recruiting**	70.1	54	64.2	113	66	167	
**Not reported**	2.6	2	11.9	21	9.1	23	
**Recruiting**	24.7	19	23.3	41	23.7	60	
**Terminated**	1.3	1	0	0	0.4	1	
**Total**	100	77	100	176	100	253	

For peer review only

Planned, ongoing and completed tuberculosis treatment trials in Brazil, Russia, India, China, and South Africa: A 2019 cross-sectional descriptive analysis

Fisher exact test is used to calculate the p value. There is significant difference when comparing recruitment status between recruitment country type.

Table 6: Two-way table comparing funding sources between recruitment country types

Funding source grouped	Multiple countries		Single countries		Total		P value*
	%	No.	%	No.	%	No.	0.00
**Local_Organisation**	11.7	9	31.3	55	25.3	64	
**International_organisation**	46.8	36	12.5	22	22.9	58	
**multifunding**	9.1	7	6.8	12	7.5	19	
**Industry**	20.8	16	15.9	28	17.4	44	
**University**	5.2	4	10.2	18	8.7	22	
**Hospital**	1.3	1	7.4	13	5.5	14	
**Self_funding**	1.3	1	8	14	5.9	15	
**Other**	3.9	3	8	14	6.7	17	
**Total**	100	77	100	176	100	253	

*chi 2 used to calculate the p value. There are significant differences when comparing funding sources between recruitment country types.

Table 7: Two-way table comparing primary outcomes of published trials between recruitment country types.

Primary Outcome	Multiple countries		Single countries		Total		P value
	%	No.	%	No.	%	No.	0.029
**Yes**	81.4	35	60.3	35	69.3	70	
**No**	18.6	8	39.7	23	30.7	31	
**Total**	100	43	100	58	100	101	

Fisher exact test is used to calculate the p value. There is significant difference when comparing primary outcome of published studies only between recruitment country type.

For peer review only

Planned, ongoing and completed tuberculosis treatment trials in Brazil, Russia, India, China, and South Africa: A 2019 cross-sectional descriptive analysis

SECTION B: Survival analysis.

This graph shows the overall Kaplan-Meier survival estimate, with a median publication time of 25 months.

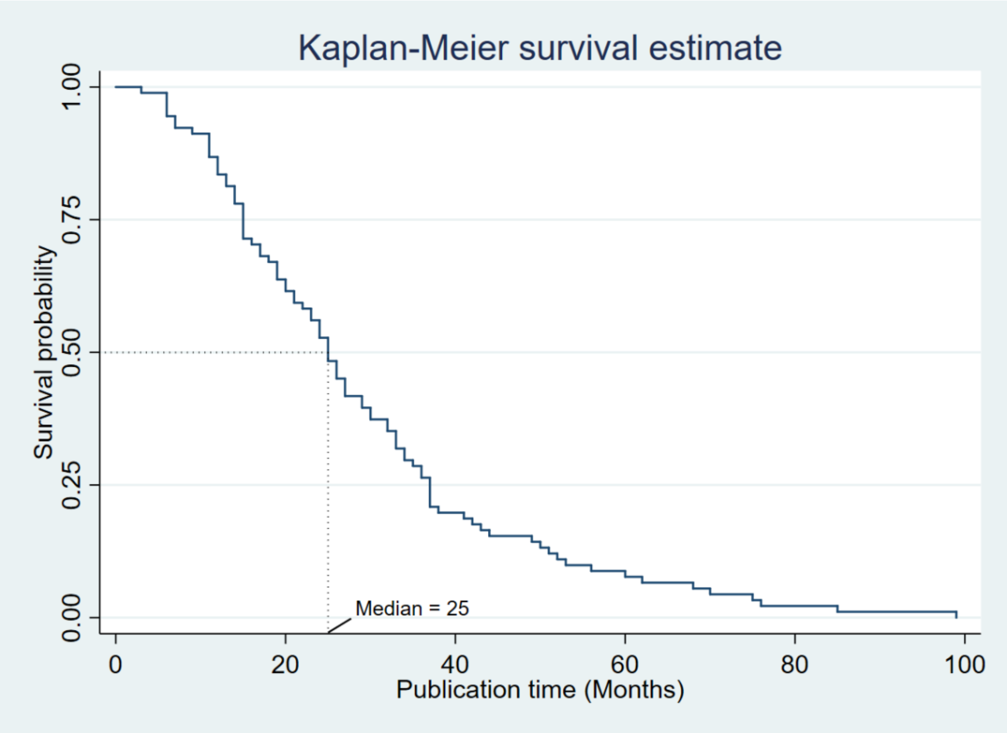

Figure 1: This graph shows the overall Kaplan-Meier survival estimate for single country with a median publication time of 26 months.

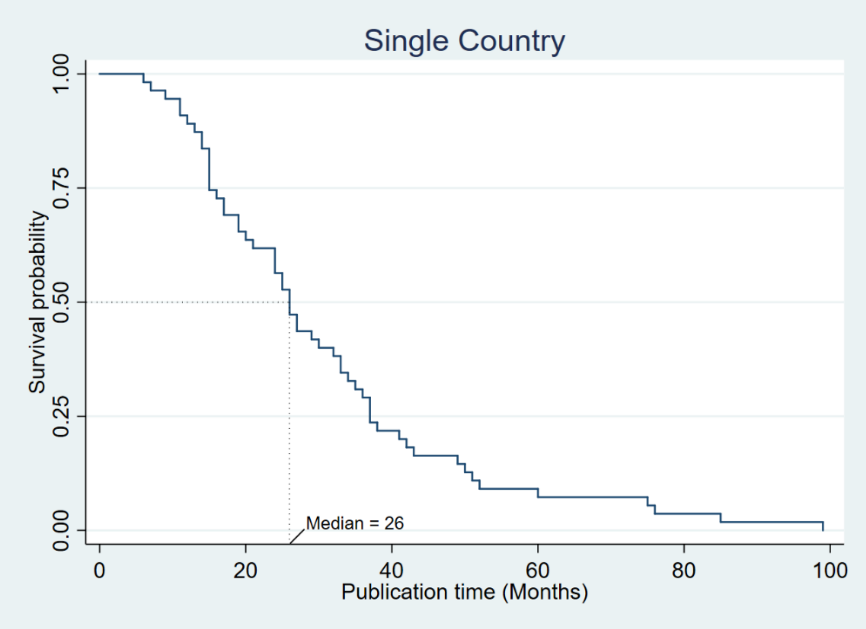

For peer review only - http://bmjopen.bmj.com/site/about/guidelines.xhtml

Planned, ongoing and completed tuberculosis treatment trials in Brazil, Russia, India, China, and South Africa: A 2019 cross-sectional descriptive analysis

Figure 2: This graph shows the overall Kaplan-Meier survival estimate for multinational studies with a median publication time of 23 months. This shows that multinational studies takes shorter time to publish as compared with single country study.

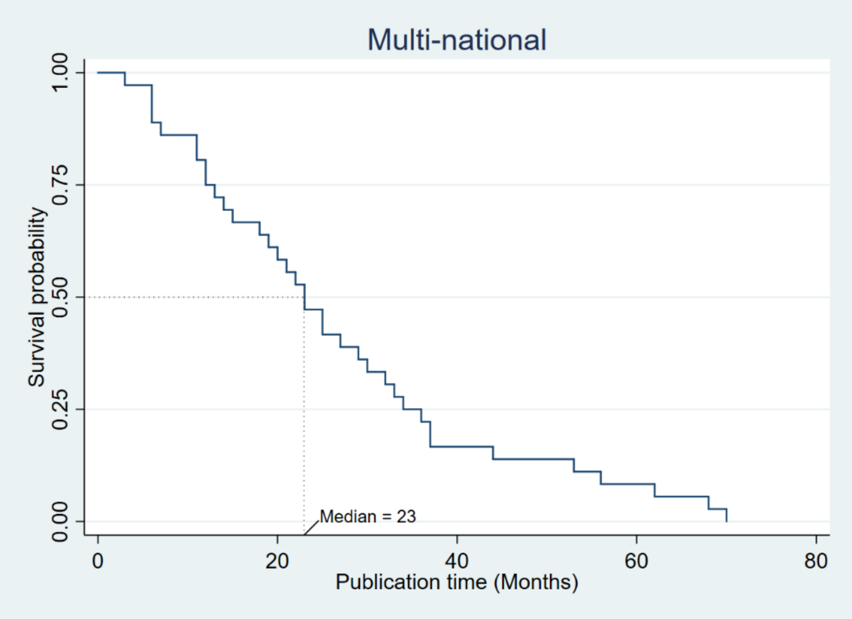

Figure 3: This graph shows the overall Kaplan-Meier survival estimate for India trial location with a median publication time of 29 months.

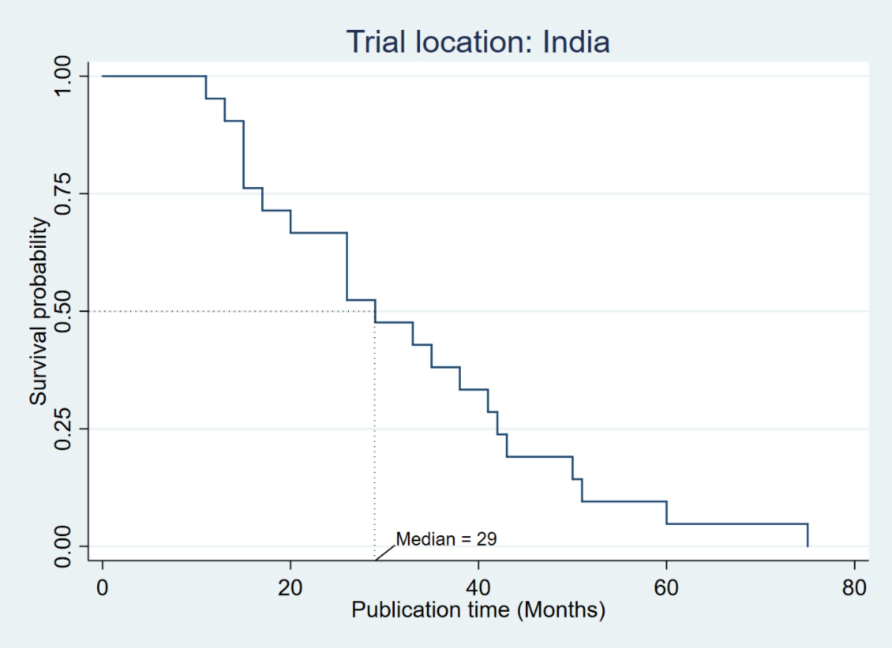

Planned, ongoing and completed tuberculosis treatment trials in Brazil, Russia, India, China, and South Africa: A 2019 cross-sectional descriptive analysis

Figure 4: This graph shows the overall Kaplan-Meier survival estimate for China trial location with a median publication time of 25 months.

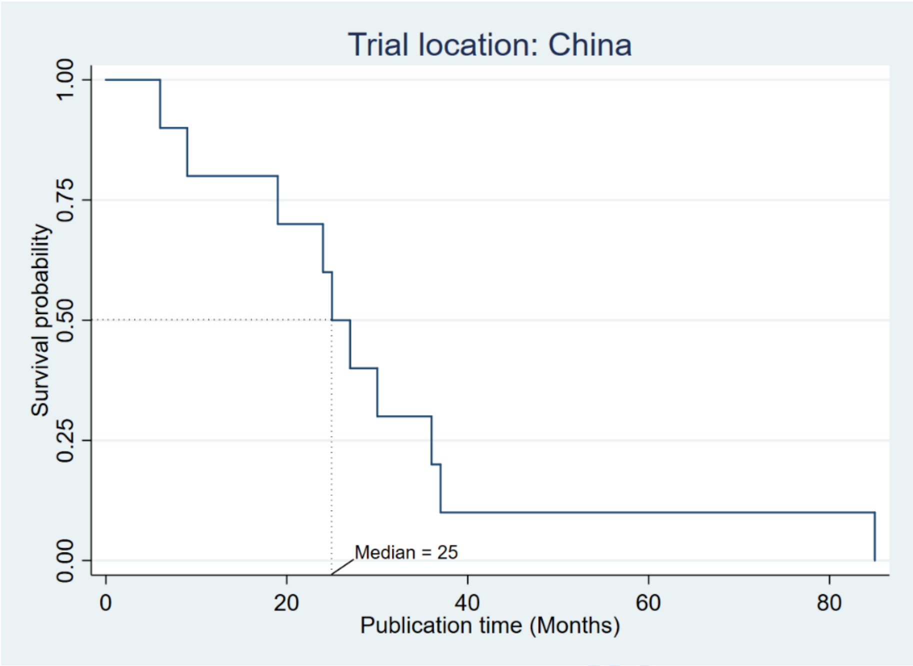

Figure 5: This graph shows the overall Kaplan-Meier survival estimate for Multi-countries trial location with a median publication time of 23 months.

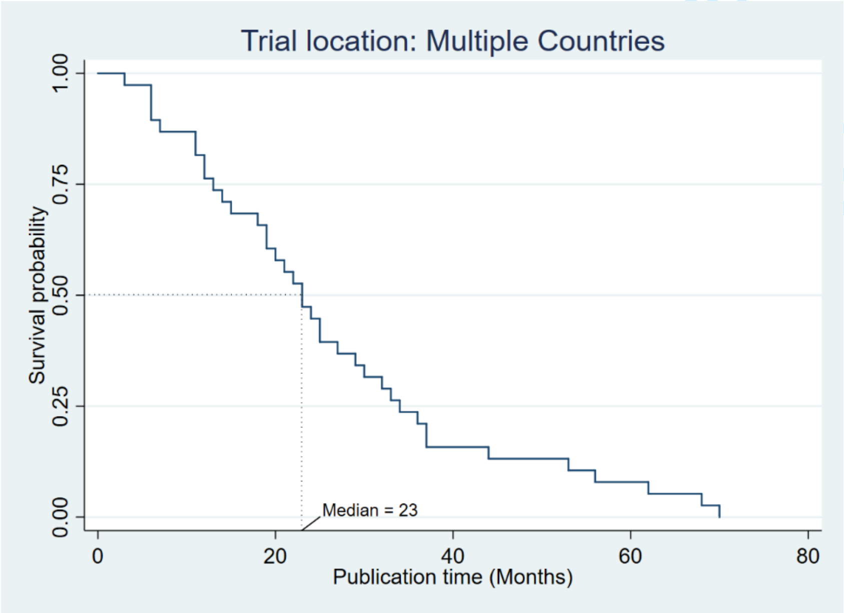

Figure 6: This graph shows the overall Kaplan-Meier survival estimate for combine Brazil, Russia and South Africa trial location with a median publication time of 24 months.

Planned, ongoing and completed tuberculosis treatment trials in Brazil, Russia, India, China, and South Africa: A 2019 cross-sectional descriptive analysis

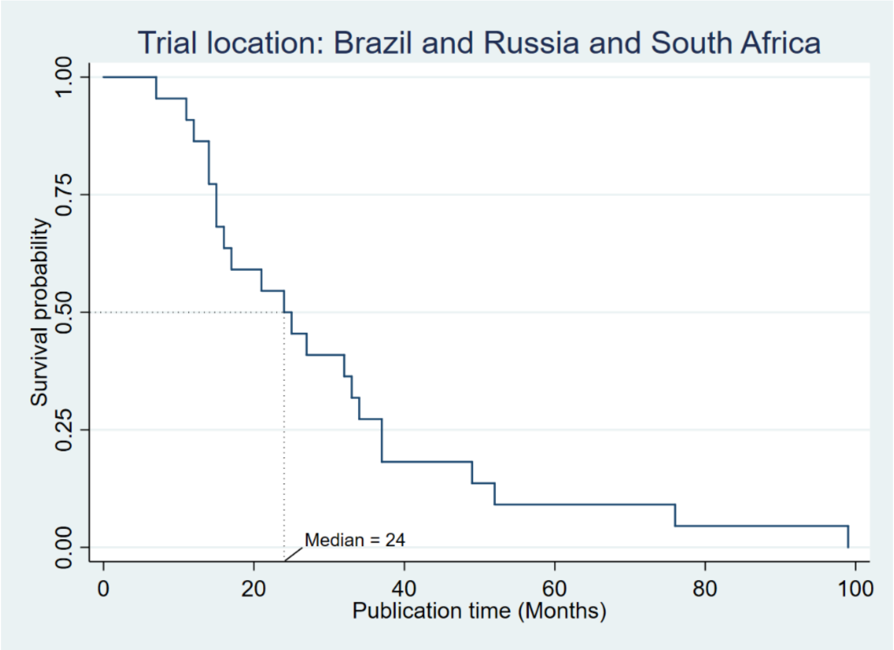

Figure 7: Multi-countries trial location studies takes shorter time to publish as compared to the other trial locations.

For peer review only - http://bmjopen.bmj.com/site/about/guidelines.xhtml

Planned, ongoing and completed tuberculosis treatment trials in Brazil, Russia, India, China, and South Africa: A 2019 cross-sectional descriptive analysis

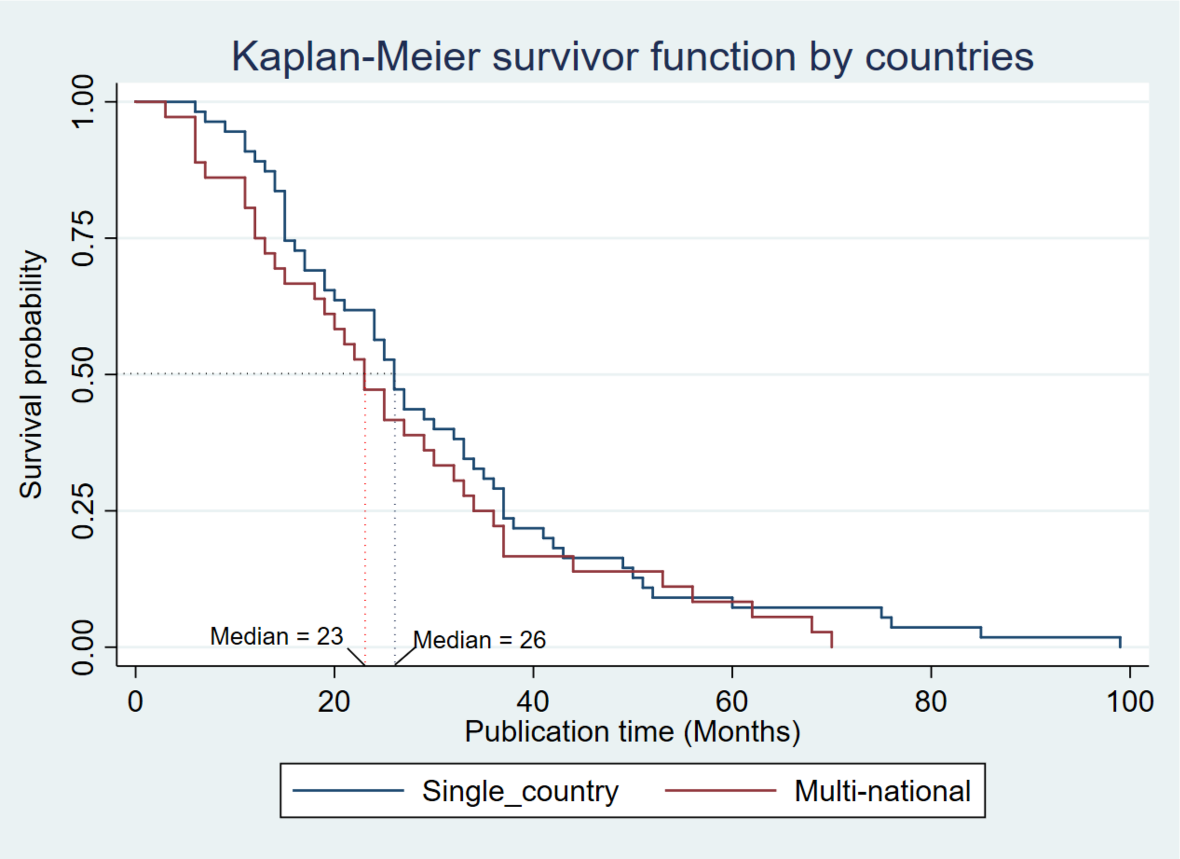

Figure 8: graph showing publication time for single country verse multi-national country.

Table 8: Test For Equality Between Groups

	n (%)	Logrank test	Wilcoxon test
Countries			
Single	171 (70)	0.3117	0.2617
Multi-national	73 (30)		
Trial country			
China	52 (21)	0.6789	0.5012
India	51 (21)		
Multiple Countries	73 (30)		
Brazil, Russia & SA	68 (28)		
Registrationstat			
Not reported	9 (21)	0.413	0.3558
Prospective	64 (156)		
Retrospective	27 (67)		

For peer review only - http://bmjopen.bmj.com/site/about/guidelines.xhtml

Planned, ongoing and completed tuberculosis treatment trials in Brazil, Russia, India, China, and South Africa: A 2019 cross-sectional descriptive analysis

The table 1, compares the curves between the group for each variable used for survival analysis. We used Log-rank test and Wilcoxon test to compares whether the publication time between the groups is different in average. We see that there is no significance difference between the groups. For example, there is no significance difference between publication time for single country verse multi-national country.

For peer review only

STROBE Statement—Checklist of items that should be included in reports of ***cross-sectional studies***

	Item No	Recommendation	Page No
**Title and abstract**	1	(*a*) Indicate the study's design with a commonly used term in the title or the abstract	6
		(*b*) Provide in the abstract an informative and balanced summary of what was done and what was found	2
**Introduction**			
Background/rationale	2	Explain the scientific background and rationale for the investigation being reported	4-6
Objectives	3	State specific objectives, including any prespecified hypotheses	6
**Methods**			
Study design	4	Present key elements of study design early in the paper	6
Setting	5	Describe the setting, locations, and relevant dates, including periods of recruitment, exposure, follow-up, and data collection	6
Participants	6	(*a*) Give the eligibility criteria, and the sources and methods of selection of participants	7
Variables	7	Clearly define all outcomes, exposures, predictors, potential confounders, and effect modifiers. Give diagnostic criteria, if applicable	-
Data sources/ measurement	8*	For each variable of interest, give sources of data and details of methods of assessment (measurement). Describe comparability of assessment methods if there is more than one group	8-9
Bias	9	Describe any efforts to address potential sources of bias	-
Study size	10	Explain how the study size was arrived at	9
Quantitative variables	11	Explain how quantitative variables were handled in the analyses. If applicable, describe which groupings were chosen and why	9
Statistical methods	12	(*a*) Describe all statistical methods, including those used to control for confounding	-
		(*b*) Describe any methods used to examine subgroups and interactions	-
		(*c*) Explain how missing data were addressed	-
		(*d*) If applicable, describe analytical methods taking account of sampling strategy	8-9
		(*e*) Describe any sensitivity analyses	-
**Results**			
Participants	13*	(a) Report numbers of individuals at each stage of study—eg numbers potentially eligible, examined for eligibility, confirmed eligible, included in the study, completing follow-up, and analysed	9
		(b) Give reasons for non-participation at each stage	-
		(c) Consider use of a flow diagram	9
Descriptive data	14*	(a) Give characteristics of study participants (eg demographic, clinical, social) and information on exposures and potential confounders	7-8
		(b) Indicate number of participants with missing data for each variable of interest	9
Outcome data	15*	Report numbers of outcome events or summary measures	15
Main results	16	(*a*) Give unadjusted estimates and, if applicable, confounder-adjusted estimates and their precision (eg, 95% confidence interval). Make clear which confounders were adjusted for and why they were included	-

For peer review only

		(*b*) Report category boundaries when continuous variables were categorized	9
		(*c*) If relevant, consider translating estimates of relative risk into absolute risk for a meaningful time period	-
Other analyses	17	Report other analyses done—eg analyses of subgroups and interactions, and sensitivity analyses	-
**Discussion**			
Key results	18	Summarise key results with reference to study objectives	16-18
Limitations	19	Discuss limitations of the study, taking into account sources of potential bias or imprecision. Discuss both direction and magnitude of any potential bias	18
Interpretation	20	Give a cautious overall interpretation of results considering objectives, limitations, multiplicity of analyses, results from similar studies, and other relevant evidence	15-18
Generalisability	21	Discuss the generalisability (external validity) of the study results	17-18
**Other information**			
Funding	22	Give the source of funding and the role of the funders for the present study and, if applicable, for the original study on which the present article is based	19

*Give information separately for exposed and unexposed groups.

Note: An Explanation and Elaboration article discusses each checklist item and gives methodological background and published examples of transparent reporting. The STROBE checklist is best used in conjunction with this article (freely available on the Web sites of PLoS Medicine at http://www.plosmedicine.org/, Annals of Internal Medicine at http://www.annals.org/, and Epidemiology at http://www.epidem.com/). Information on the STROBE Initiative is available at www.strobe-statement.org.

For peer review only
